# 3D Perovskite Passivation with a Benzotriazole-Based 2D Interlayer for High-Efficiency Solar Cells

**DOI:** 10.1021/acsaem.3c00101

**Published:** 2023-03-27

**Authors:** Alessandro Caiazzo, Arthur Maufort, Bas T. van Gorkom, Willemijn H. M. Remmerswaal, Jordi Ferrer Orri, Junyu Li, Junke Wang, Wouter T. M. van Gompel, Kristof Van Hecke, Gunnar Kusch, R. A. Oliver, Caterina Ducati, Laurence Lutsen, Martijn M. Wienk, Samuel D. Stranks, Dirk Vanderzande, René A. J. Janssen

**Affiliations:** † Molecular Materials and Nanosystems and Institute of Complex Molecular Systems Eindhoven University of Technology, P.O. Box 513, 5600 MB Eindhoven, The Netherlands; ‡ Institute for Materials Research (IMO-IMOMEC), Hybrid Materials Design, Hasselt University, Martelarenlaan 42, B-3500 Hasselt, Belgium; § Cavendish Laboratory, University of Cambridge, Cambridge CB3 0HE, United Kingdom; ∥ Department of Materials Science and Metallurgy, University of Cambridge, Cambridge CB3 0FS, United Kingdom; ⊥ Department of Chemical Engineering and Biotechnology, University of Cambridge, Cambridge CB3 0HE, United Kingdom; # XStruct, Department of Chemistry, Ghent University, Krijgslaan 281-S3, B-9000 Ghent, Belgium; ∇ Dutch Institute for Fundamental Energy Research, De Zaale 20, 5612 AJ Eindhoven, The Netherlands

**Keywords:** 2D perovskites, passivation, solar cells, FAPbI_3_, benzotriazole

## Abstract

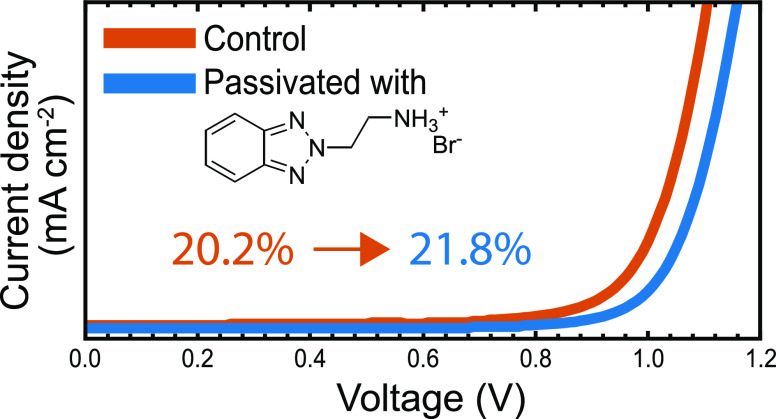

2H-Benzotriazol-2-ylethylammonium bromide and iodide and its difluorinated derivatives are synthesized and employed as interlayers for passivation of formamidinium lead triiodide (FAPbI_3_) solar cells. In combination with PbI_2_ and PbBr_2_, these benzotriazole derivatives form two-dimensional (2D) Ruddlesden–Popper perovskites (RPPs) as evidenced by their crystal structures and thin film characteristics. When used to passivate n–i–p FAPbI_3_ solar cells, the power conversion efficiency improves from 20% to close to 22% by enhancing the open-circuit voltage. Quasi-Fermi level splitting experiments and scanning electron microscopy cathodoluminescence hyperspectral imaging reveal that passivation provides a reduced nonradiative recombination at the interface between the perovskite and hole transport layer. Photoluminescence spectroscopy, angle-resolved grazing-incidence wide-angle X-ray scattering, and depth profiling X-ray photoelectron spectroscopy studies of the 2D/three-dimensional (3D) interface between the benzotriazole RPP and FAPbI_3_ show that a nonuniform layer of 2D perovskites is enough to passivate defects, enhance charge extraction, and decrease nonradiative recombination.

## Introduction

Perovskite solar cells based on formamidinium lead iodide (FAPbI_3_) as an active layer have recently become a standard baseline to reach a high power conversion efficiency (PCE).^1^ This material introduces new challenges in processing, mainly related to stabilizing the photoactive α-phase at room temperature, as the photoinactive δ-phase is thermodynamically more stable.^2,3^ Careful use of additive engineering and a variety of processing methods help to stabilize three-dimensional (3D) perovskite FAPbI_3_ in a tetragonal phase with slight octahedral tilting,^4^ paving the way for solar cells with narrower band gap compared to MAPbI_3_ and with extremely low voltage losses.^5−8^ Currently, the state-of-the-art FAPbI_3_ films make use of methylammonium chloride (MACl) as additive, with other notable cases using methylene diammonium dichloride.^1,9−12^ Such additives induce octahedral tilting, which inhibits the transformation to photoinactive δ-phase.^4^ Additionally, these compounds are volatile; thus, they do not remain in the film after thermal annealing and do not impact the optical band gap with the presence of methylammonium (MA) or chloride (Cl) ions.

As widely mentioned in the literature, the interface between the perovskite and charge transport layers is of crucial importance to reduce nonradiative recombination losses and achieve high open-circuit voltage (*V*_OC_) with respect to the radiative limit.^13,14^ More specifically, in solar cells with n–i–p configurations, it has been shown that the interface between the perovskite and the 2,2′,7,7′-tetrakis[*N*,*N*-di(4-methoxyphenyl)amino]-9,9′-spirobifluorene (Spiro-OMeTAD) hole-transporting layer can be passivated with organic spacers that are able to form two-dimensional (2D) or quasi-2D perovskites.^15−19^ These lower dimensional perovskites consist of inorganic layers of lead halide octahedra sandwiched between large organic spacers, which are too bulky to fit into octahedral voids to form a 3D perovskite.^20^ Despite the large number of studies published on this topic, no consensus has been reached on the nature of the 2D interlayer found at the perovskite/Spiro-OMeTAD interface, its uniformity, and the mechanism behind commonly reported performance enhancement. Moreover, one of the main advantages of employing 2D perovskites is their chemical variability since they can be formed ideally with many bulky organic cations leading to different material properties.^21^ Despite this, most studies focus only on a handful of spacers, often based on butylammonium (BA), octylammonium (OA), and phenethylammonium (PEA). Functionalization of PEA with fluorine atoms and subsequent passivation of 3D perovskites have also been shown to improve solar cell PCE and stability toward humidity, mostly by making the film surface more hydrophobic and less subject to the impact of moisture.^22^ Exploration of new spacers is however limited, even though it can potentially lead to impactful discoveries.

In this work, we explore the use of benzotriazole derivatives as new spacers to form 2D perovskites and we employ them as interlayers in solar cells with FAPbI_3_-rich perovskite films as an active layer. A novel, easy-to-synthesize, and easy-to-functionalize benzotriazole derivative is proposed as an organic cation to form Ruddlesden–Popper perovskites (RPPs) and to passivate 3D perovskites. With a simple passivation strategy, the PCE of solar cells improved from about 20% to almost 22% by enhancing the *V*_OC_. Furthermore, by characterizing the 3D/2D heterostructure, we elucidated the formation mechanism of the benzotriazole-based RPP and found that a nonuniform layer of 2D perovskites is enough to passivate defects, enhance charge extraction, and decrease nonradiative recombination at the hole transport layer interface.

## Results and Discussion

### Synthesis of Benzotriazole Derivatives

**Figure 1:**
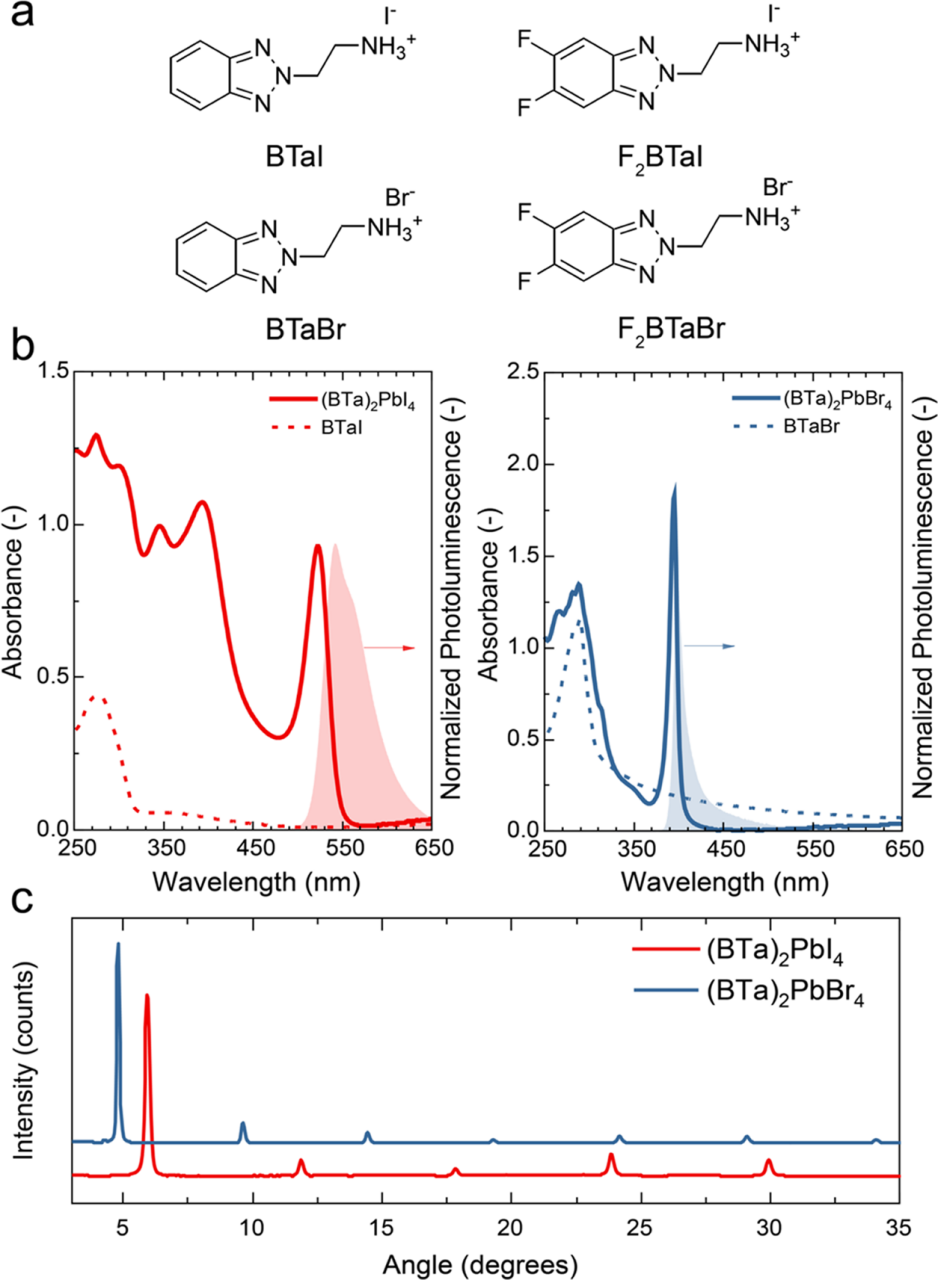
(a) Chemical structure of benzotriazole derivatives used in this study. (b) Ultraviolet–visible (UV–vis) absorption (solid line) and photoluminescence (PL) emission spectra (shaded area) of (BTa)_2_PbI_4_ (left) and (BTa)_2_PbBr_4_ (right) thin films. Dashed lines represent absorption spectra of thin films of BTaI and BTaBr salts. The PL emission spectra of (BTa)_2_PbI_4_ and (BTa)_2_PbBr_4_ were obtained with excitation at 430 and 300 nm, respectively. (c) X-ray diffraction (XRD) patterns of the same perovskites as in panel (b).

Benzotriazole (BTa) is a versatile aromatic building block that allows for facile derivatization for application in perovskite-based solar cells. It is a weak electron acceptor that has already featured in donor–acceptor type conjugated polymers for organic photovoltaics in the past.^23,24^ A benzotriazole derivative has also been recently used for passivation purposes on perovskite solar cells.^25^ The benzotriazole unit can be synthesized conveniently from an *o*-phenylenediamine and sodium nitrite and, since many of these diamines are commercially available, it is straightforward to introduce substituents. For this work, we targeted both pristine benzotriazole and a difluorinated derivative. Through a Mitsunobu reaction, both units were functionalized with a two-carbon alkyl chain containing a *tert*-butyloxycarbonyl (Boc)-protected amino group. Finally, using either HBr or HI, the amine was deprotected and converted into an ammonium bromide or iodide, respectively. This reaction yields BTaBr and BTaI out of the pristine unit and F_2_BTaBr and F_2_BTaI out of the fluorinated unit (Figure 1a). Details on the synthesis procedures and characterization can be found in the Experimental Section (Supporting Information). This straightforward three-step process, consisting of core formation, alkylation, and salt formation, can be applied to various *o*-phenylenediamine derivatives and any alkyl tail lengths, and hence, a multitude of tailored benzotriazole salts can be synthesized. This opens the possibility for benzotriazole derivatives to be used as novel versatile building blocks for (quasi-) 2D perovskites.

To verify whether these synthesized benzotriazole salts can be used as organic cations to obtain 2D perovskites, we spin-coated thin films from precursor solutions containing a benzotriazole salt and PbI_2_ or PbBr_2_ in a 2:1 molar ratio. Figure 1b summarizes the optical properties of lead-based 2D perovskite thin films of two of the benzotriazole salts. Excitonic absorption peaks are located at 523 and 395 nm for (BTa)_2_PbI_4_ and (BTa)_2_PbBr_4_, respectively. Similar absorption peaks are reported for the fluorinated derivatives of the salts (Figure S1, Supporting Information). These wavelengths match with typical literature values for 2D lead iodide and lead bromide perovskites,^26−28^ which indicates that 2D perovskite self-assembly was successful with all salts. The UV–vis absorption spectra of (BTa)_2_PbI_4_ and (F_2_BTa)_2_PbI_4_ also clearly show excitations to higher Rydberg states at about 350 and 400 nm.^29^ Additionally, below 320 nm, the organic layer (benzotriazole unit) contributes to the absorption spectrum. The excitonic photoluminescence (PL) peaks are Stokes-shifted with respect to their corresponding absorption peaks, as expected.^24^ The PL spectra of (BTa)_2_PbI_4_ and (F_2_BTa)_2_PbI_4_ perovskites also show a shoulder at longer wavelengths. The appearance of dual emission peaks for films of hybrid perovskites can arise from self-absorption effects and thin-film cavity effects via interference.^30,31^ Following the work of van der Pol et al.,^31^ we investigated the influence of the perovskite film thickness by varying the spin coating speed and found that the intensity ratio of the emission peaks indeed depends on the film thickness, with the long-wavelength shoulder becoming more pronounced for thicker films (Figure S2, Supporting Information). This is a strong indication for cavity effects as the origin of the shoulder.^31^

The phase purity of the perovskite thin films was studied by X-ray diffraction (XRD), which clearly shows the (0 0 2*l*) reflection (*l* = 1–5 and 1–7) characteristic for a 2D perovskite grown with a preferential orientation of PbX_6_ sheets parallel to the surface (Figure 1c).^32,33^ By using Bragg’s law and the position of the first-order reflections, a *d*-spacing between the sheets of 14.9 and 18.4 Å was calculated for (BTa)_2_PbI_4_ and (BTa)_2_PbBr_4_. Similar spacings of 15.0 and 18.7 Å were found for the 2D perovskites containing the fluorinated salts (Figure S3, Supporting Information). To further elucidate the perovskite crystal structure, single crystals of all four perovskites were grown. Single crystals were obtained via an optimized solvent conversion-induced rapid crystallization method reported in the literature.^34^ Single-crystal structures are shown in Figure S4 (Supporting Information). Interestingly, the crystal structures of (BTa)_2_PbI_4_ and (F_2_BTa)_2_PbI_4_ contain organic bilayers of interdigitating benzotriazole cores, whereas (BTa)_2_PbBr_4_ and (F_2_BTa)_2_PbBr_4_ have an organic bilayer, where the benzotriazole cores of each part of the bilayer do not interdigitate, as is more frequently encountered in the literature for large organic cations.^26,28,35^ In each case, the *d*-spacing found in the thin-film XRD matches with the spacing between the PbX_6_ sheets inferred from the single-crystal structure. For (BTa)_2_PbI_4_ and (F_2_BTa)_2_PbI_4_ that both crystalize in the same orthorhombic space group, *d* = *b*/2, while for (BTa)_2_PbBr_4_ and (F_2_BTa)_2_PbBr_4_ that crystallize in different monoclinic space groups, *d* = *a*/2 and *d* = *a*, respectively (Figure S4, Supporting Information). This shows that the crystal structure in the spin-coated films matches with that of the solution-grown single crystals. We hypothesize that the smaller size of PbBr_6_ octahedrons compared to PbI_6_ precludes the interdigitation of the BTa and F_2_BTa units in the unit cell.^28^ A separate detailed structural study combined with computational modeling is ongoing to fully elucidate these aspects.

### FAPbI_3_ Passivation

**Figure 2:**
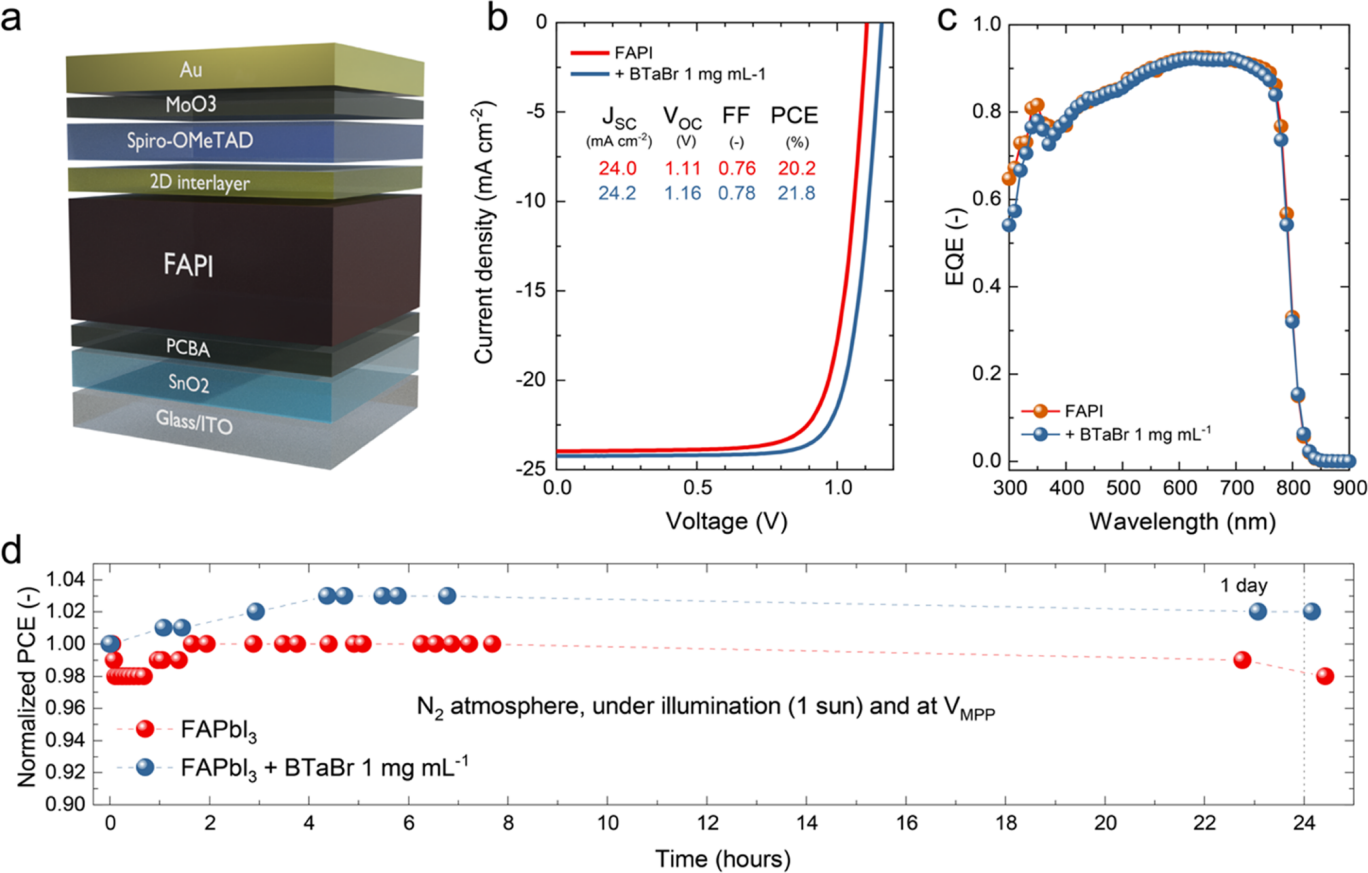
(a) Device stack for n–i–p perovskite solar cells used in this study. (b) *J*–*V* curves for FAPbI_3_ solar cells without and with BTaBr (1 mg mL^–1^) passivation. (c) External quantum efficiency (EQE) spectra for the same devices as in panel (b). (d) Stability measurement under a N_2_ atmosphere and under illumination at the maximum power point voltage (*V*_MPP_) for the same devices as in panels (b) and (c).

Once confirmed that the benzotriazole derivatives are suitable to form 2D perovskites, we employed these molecules as interlayers in solar cells in the n–i–p configuration with FAPbI_3_ 3D perovskites as an active layer. 2D perovskites at the perovskite/hole transport layer (HTL) interface should provide a passivation effect and lead to optimized band alignment, according to previous reports.^18^ The device stack is illustrated in Figure 2a. On top of indium tin oxide (ITO) as transparent conductive oxide, a SnO_2_ layer passivated by a monolayer of [6,6]-phenyl-C_61_-butyric acid (PCBA) is used as an electron-transport layer (ETL),^36^ Spiro-OMeTAD is used as a hole transport layer (HTL), and a bilayer of MoO_3_/Au as top contact. FAPbI_3_ was processed according to a two-step spin coating procedure, where the deposition of the first layer of PbI_2_ is followed by the organic components (formamidinium iodide (FAI) and methylammonium chloride (MACl)) and thermal annealing in air. The detailed procedures are described in the Experimental Section (Supporting Information). Benzotriazole salts were spin-coated from solutions of 1 and 10 mg mL^–1^ in 2-propanol, i.e., concentrations commonly reported in the literature, onto FAPbI_3_. All four benzotriazole derivatives (fluorinated, nonfluorinated, iodide, or bromide anion) were used as the passivation layer, with BTaBr providing the best results in terms of solar cell performance and reproducibility (Figure S5, Supporting Information). As a result, we decided to employ BTaBr for the rest of the study.

As shown in the current–density/voltage (*J*–*V*) curves in Figure 2b, devices without passivation layer display good photovoltaic performances and reach 20.2% PCE in the champion device. Passivation of FAPbI_3_ with diluted BTaBr (1 mg mL^–1^) significantly enhances the *V*_OC_ of the devices from 1.11 to 1.16 V and slightly improves the short-circuit current density (*J*_SC_) and fill factor (FF). As summarized in Table 1, FAPbI_3_ solar cells passivated with BTaBr reach a PCE of 21.8%. The improved performance of BTaBr passivation is reproducible over several devices, as displayed in Figure S6 (Supporting Information), and is statistically significant (one-sample *t*-test, *p*-value <0.001). Based on this statistical data, average photovoltaic parameters are reported in Table 1. When BTaBr passivation is applied using more concentrated solutions (10 mg mL^–1^), the solar cells show a critical loss in FF and overall worse photovoltaic performances because of poor charge extraction, for reasons that will be described below. We compared the photovoltaic performance of FAPbI_3_ passivated with either BTaBr or PEAI, as a more conventional organic spacer, and observed a similar increase in *V*_OC_ compared to the reference device, but a slightly higher PCE because of the enhanced FF when using BTaBr (Figure S7, Supporting Information). Figure 2c shows the external quantum efficiency (EQE) spectra of the solar cells. All devices possess a high EQE with peak values above 95% and an integrated current density (∼24 mA cm^–2^) that closely matches the one obtained via *J*–*V* measurements. The passivated device shows a slightly higher EQE compared to the reference. The EQE drops in the green region of the spectrum (below 550 nm), which prevents the devices from reaching *J*_SC_ > 25 mA cm^–2^ and PCE > 22%. We attribute this EQE loss, at least in part, to absorption by the ITO bottom electrode (Figure S8, Supporting Information).

**Table 1:** Average Photovoltaic Parameters of Nonpassivated and BTaBr-Passivated FAPbI_3_ Solar Cells^a^

passivation	*J*_SC_ (mA cm^–2^)	*V*_OC_ (V)	FF (-)	PCE (%)
none	24.05 ± 0.66 (24.00)	1.03 ± 0.04 (1.11)	0.78 ± 0.03 (0.76)	19.4 ± 0.96 (20.2)
BTaBr 1 mg mL^–1^	24.20 ± 0.65 (24.16)	1.10 ± 0.03 (1.16)	0.78 ± 0.02 (0.78)	20.6 ± 0.68 (21.8)
BTaBr 10 mg mL^–1^	(23.80)	(1.11)	(0.60)	(15.8)

a Device layout: ITO/SnO_2_/PCBA/FAPbI_3_/passivation layer/Spiro-OMeTAD/MoO_3_/Au. Champion cell values are in parenthesis. The number of cells fabricated are *N* = 10 and *N* = 17 for the reference device and passivated with BTaBr 1 mg mL^–1^, respectively. For the 10 mg mL^–1^ BTaBr passivation, no statistics are reported as few devices were fabricated. Hysteresis indexes for the champion devices are 0.17 and 0.07 for nonpassivated and BTaBr-passivated, respectively.

A preliminary study of the stability of the solar cells under illumination held at a maximum power point voltage (*V*_MPP_) is shown in Figure 2d. Cells with and without passivation are stable under ∼24 h continuous illumination. FAPbI_3_ retained 98% of the initial PCE, whereas FAPbI_3_ + BTaBr 1 mg mL^–1^ was slightly more efficient (by about 4%) than at the beginning of the measurement. Such an increase might be related to an increase in *V*_OC_ over time, which would shift the *V*_MPP_. Changes of MPP are not taken into account during the measurement, as the *V*_MPP_ is chosen according to the first *J*–*V* measurement and not subsequently tracked.

### Passivation Effect of BTaBr

**Figure 3:**
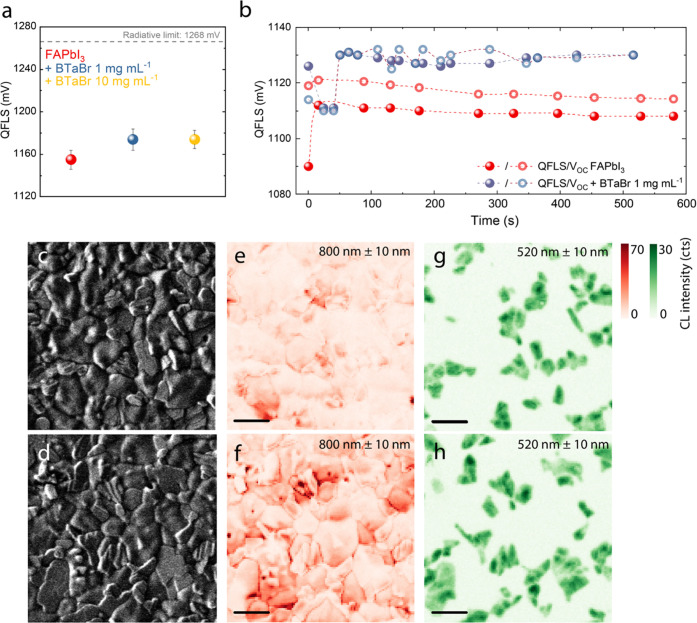
(a) QFLS calculated from absolute PL for neat perovskite films with and without passivation. (b) Time-dependent QFLS and *V*_OC_ for full devices without and with diluted BTaBr passivation. (c, d) Scanning electron microscopy (SEM) images of FAPbI_3_ (c) and FAPbI_3_ + BTaBr (1 mg mL^–1^) (d). (e–h) Cathodoluminescence (CL) emission intensity as shown in the SE images in panels (c) and (d). Wavelength of emission is filtered at 800 ± 10 nm (e, f) and 520 ± 10 nm (g, h) to visualize emission from the 3D perovskite and PbI_2_, respectively. Scale bar is 1 μm for all images.

The *V*_OC_ = 1.16 V of the best BTaBr-passivated FAPbI_3_ solar cells represents 91% of the radiative limit (1268 mV based on a band gap of 1.55 eV, calculated via inflection point in EQE), indicating small nonradiative losses. To elucidate the effect of the passivation layer on the *V*_OC_, we employed absolute photoluminescence (PL) measurements to evaluate differences in the quasi-Fermi level splitting (QFLS) between nonpassivated and passivated FAPbI_3_ films. The QFLS of the nonpassivated films is 1161 ± 8 mV, i.e., ∼100 mV below the radiative limit (1268 mV based on a band gap of 1.54 eV) (Figure 3a). Passivation by BTaBr leads to a small increase (∼4 mV) of the QFLS to 1165 ± 7 mV. The enhancement in QFLS is less than the increase in average *V*_OC_ measured in devices (∼70 mV, Table 1). This suggests that the enhanced *V*_OC_ might also be related to passivation of the perovskite/HTL interface, rather than of the perovskite film itself. To confirm this, we measured the QFLS and *V*_OC_ of full devices with and without passivation over time (Figure 3b). Montoring the *V*_OC_ and QFLS over time was necessary because when measuring perovskite/Spiro-OMeTAD films, the absolute PL signal becomes time-dependent and the peaks in the first few seconds, to then stabilize at a lower value (Figure S9, Supporting Information). Following QFLS and *V*_OC_ of complete devices over time, we observed a significant increase of both quantities for the passivated devices by about ∼20 mV (QFLS) and ∼15 mV (*V*_OC_). This suggests that BTaBr can have important passivation properties at the perovskite/HTL interface, in agreement with the consistent increase in *V*_OC_ observed in the solar cells. A similar increase in the stabilized-QFLS is observed after passivation when using FAPbI_3_/(2D)/Spiro-OMeTAD samples (Figure S9, Supporting Information).

To further investigate the passivation effect of BTaBr, we employed scanning electron microscopy cathodoluminescence (SEM-CL) hyperspectral imaging (Figure 3c–h). First, merely from a morphological point of view, new crystallites appear on the surface of FAPbI_3_ after passivation with diluted BTaBr (Figure 3c–d). Such crystallites are more clearly visible in Figure S10 (Supporting Information). No closed interlayer seems to be formed. Instead, after passivation with a more concentrated BTaBr solution (10 mg mL^–1^), a new layer appears on top of FAPbI_3_, and no perovskite grains can be observed (Figure S11, Supporting Information). Figure 3e–f displays the 3D perovskite CL emission intensity maps for the 3D perovskite FAPbI_3_, bandpass filtered at 800 ± 10 nm, before and after passivation with diluted BTaBr. For comparison, PbI_2_ emission mapping (using a bandpass filter at 520 ± 10 nm) is shown in Figure 3g,h. Both CL hyperspectral maps were acquired under the same conditions, so in each image, a more saturated coloring indicates stronger CL. Figure 3e indicates that before passivation, FAPbI_3_ exhibits a low and inhomogeneous CL emission intensity with most grains being relatively nonemissive, and few displaying bright CL emission, sometimes in the proximity of PbI_2_ grains. A more detailed image with spectral analysis of different grains is displayed in Figure S12 (Supporting Information). After passivation, the 3D perovskite CL emission becomes ∼1.5 to 3 times brighter (Figure S13, Supporting Information). Additionally, the grain boundaries display stronger emission compared to the nonpassivated film. Such an increase in emission, both intragrain and at grain boundaries after passivation, is in agreement with other reports.^37^ Interestingly, for both nonpassivated and passivated films, the grain boundaries do not seem to represent a location with enhanced nonradiative recombination but instead show a relatively high CL emission. This is likely because morphology affects the CL intensity and rougher surfaces (i.e., sharp grain boundaries) can result in brighter CL emission. Just as observed in FAPbI_3_, also the passivated layer displays a relatively high PbI_2_ emission, and sometimes, it appears that perovskite emission is stronger at the boundary between the perovskite and PbI_2_. To demonstrate this, we analyzed a passivated perovskite film with higher magnification (Figure S14, Supporting Information) and found that emission from the 3D perovskite is indeed brighter when the signal overlaps with PbI_2_ emission, which might suggest a passivation effect from PbI_2_ itself. The overlap of signals at 800 nm (FAPbI_3_) and 520 nm (PbI_2_) is dictated by the fact that SEM-CL can detect emission not only from the surface but up to a penetration depth of ∼60 nm (at 3 kV electron acceleration voltage), meaning that perovskite crystals under PbI_2_ superficial grains can be measured.

With regard to FAPbI_3_ passivated with concentrated BTaBr, despite a drastic morphology change because of the presence of a relatively thick 2D interlayer, we could again identify only bright 3D perovskite emission and emission from PbI_2_ grains (Figure S15, Supporting Information). In all films, no emission from the 2D perovskite phases could be detected. This could be caused by efficient charge or energy transfer mechanism from 2D to 3D phase, which leads to only emission at ∼800 nm being observed. No signs of beam-induced damage from the 2D emission were detected either (Figure S13, Supporting Information). Nevertheless, both QFLS and SEM-CL suggest that BTaBr has a passivating effect, which reduces nonradiative recombination and leads to stronger CL and PL emission and, as a result, an enhanced *V*_OC_.

### 2D/3D Heterostructure

**Figure 4:**
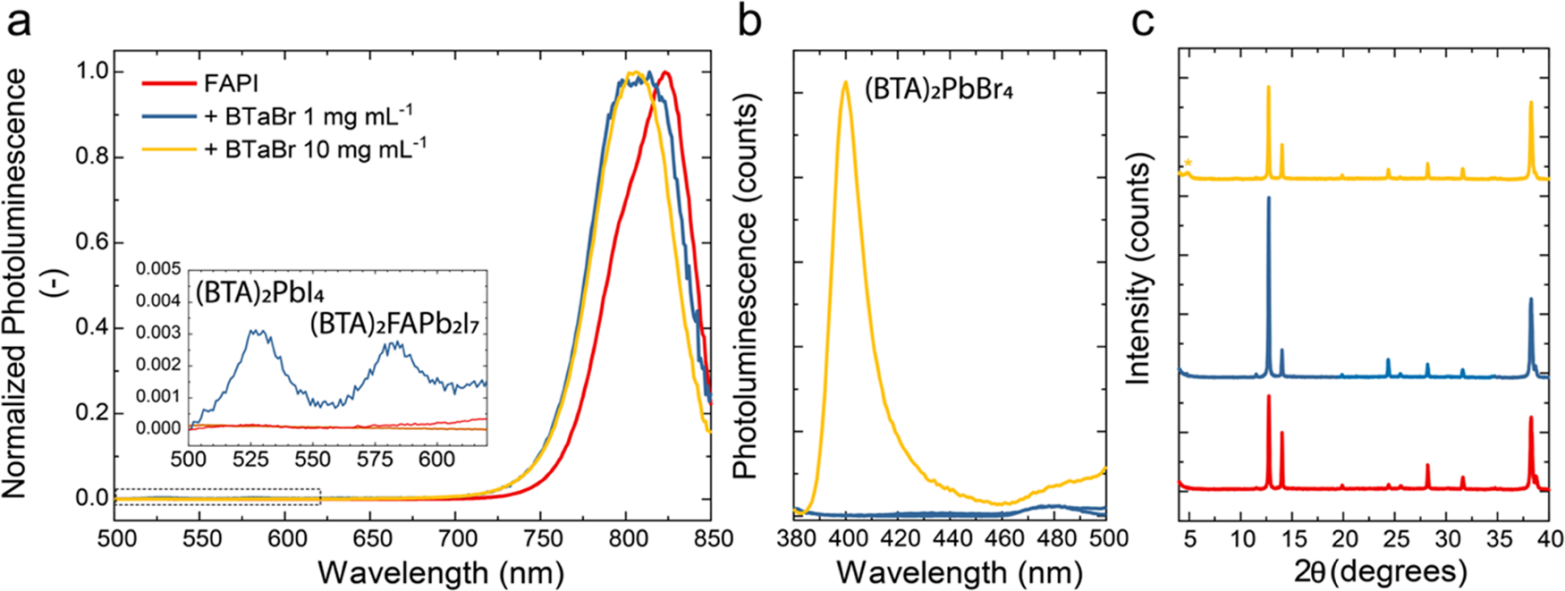
(a) PL emission spectra before and after passivation of FAPbI_3_ with BTaBr at different concentrations (1 and 10 mg mL^–1^). Inset shows the same plot but zoomed-in between 500 and 620 nm. (b) Same PL emission spectra as (a) but at lower wavelengths. (c) XRD patterns for the same films as in panel (a). The asterisk indicates (BTa)_2_PbBr_4_ diffraction peak.

Having established the improvement of solar cell performance after 2D passivation, we aimed to characterize in detail the perovskite/BTaBr heterostructure. To do so, we first employed PL spectroscopy. Emission peaks at 530 and 570 nm, corresponding to 100%-iodide *n* = 1 (2D, (BTa)_2_PbI_4_) and *n* = 2 (quasi-2D, (BTa)_2_FAPb_2_I_7_) phases, were observed after spin coating diluted BTaBr (1 mg mL^–1^) on top of FAPbI_3_ (Figure 4a). Hence, despite the use of the bromide salt for passivation, only PL from an iodide-based 2D perovskite was observed. In contrast, FAPbI_3_ films passivated with concentrated BTaBr (10 mg mL^–1^) did not display such emission peaks, indicating that no iodide-based 2D or quasi-2D perovskite phases were formed in this case. By analyzing the PL emission spectra at shorter wavelengths (Figure 4b), however, we could identify an emission peak at about 400 nm, which corresponds to (BTa)_2_PbBr_4_, as shown in Figure 1b. The main 3D perovskite emission peak is located at 820 nm, which represents a band gap energy (*E*_g_) of 1.51 eV, in line with the band gap of 1.55 eV obtained via EQE.

The XRD patterns of the 3D FAPbI_3_ perovskite, without or with BTaBr passivation, show reflections at 2θ = 14 and 28° corresponding to α-FAPbI_3_ (110) and (220) planes (Figure 4c). Small reflections at 2θ = 11.5° demonstrate the presence of negligible amounts of δ-FAPbI_3_, whereas the reflection at 2θ = 12.7° represents PbI_2_ and dominates the whole diffractogram. The XRD pattern of the FAPbI_3_/BTaBr heterostructure showed no reflections at 2θ angles lower than 10°, which are representative of the lower dimensional perovskites, after passivation with the diluted salt (Figure 4c). It is plausible that the amount of 2D perovskite phase present in these films is too small to be detected using XRD, which is less sensitive than absorption and photoluminescence spectroscopy to small amounts of low-dimensional perovskite present in a thin film.^34^ On the other hand, a peak at 2θ = 4.8° appeared after passivation with concentrated BTaBr. This peak corresponds to the (BTa)_2_PbBr_4_ phase (Figure 1c). Based on the combination of PL and XRD, we hypothesize that BTaBr first reacts with excess PbI_2_ or replaces FA from FAPbI_3_ on the surface to form (BTa)_2_PbI_4_, as seen after passivation with the diluted benzotriazole salt. Then, when the concentration of the passivating agent increases, an excess of Br ions replaces I, forming a 100%-Br 2D perovskite structure on top of the 3D perovskite. The presence of Br on the surface is further confirmed by X-ray photoelectron spectroscopy (XPS), which shows a higher amount of Br with increasing concentration of the passivation salt (Figure S16, Supporting Information).

**Figure 5:**
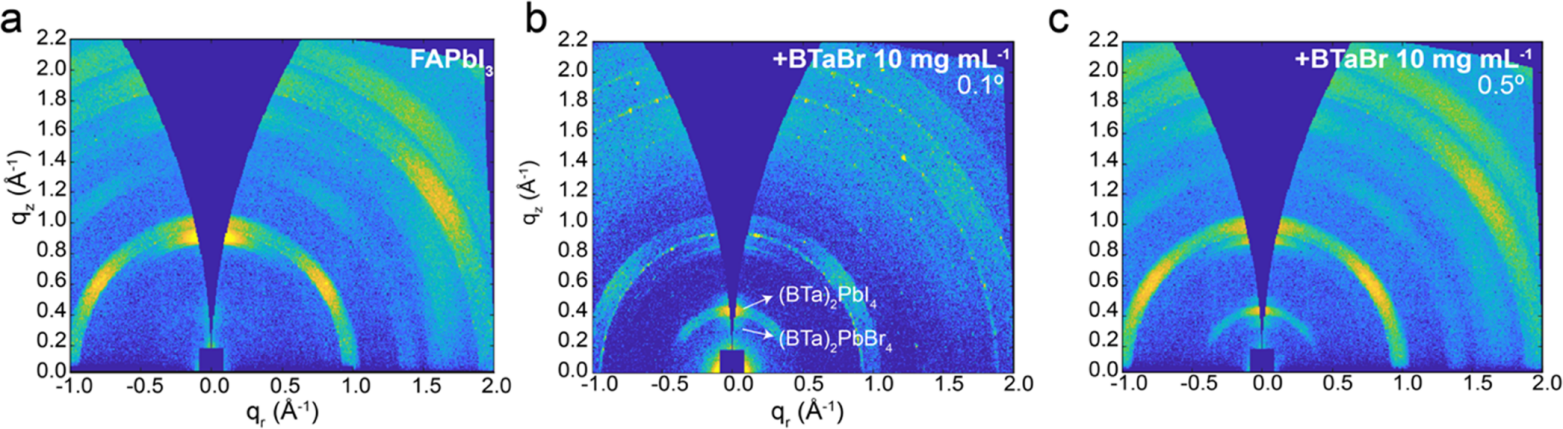
GIWAXS 2D pattern for (a) FAPbI_3_, (b) FAPbI_3_ + BTaBr 10 mg mL^–1^, 0.1° incidence angle, and (c) FAPbI_3_ + BTaBr 10 mg mL^–1^, 0.5° incidence angle.

To gain more information on the 2D/3D heterostructure and on the presence of PbI_2_ detected through XRD (Figure 4c), we employed angle-resolved grazing-incidence wide-angle X-ray scattering (AR-GIWAXS). The GIWAXS pattern of the FAPbI_3_ films (Figure 5a) shows an intense Bragg spot at *q*_*z*_ = 0.9 Å^–1^ in the out-of-plane direction, corresponding to PbI_2_ crystallites with a significant degree of preferential orientation, consistent with the strong reflection at 2θ = 12.7° in the diffractogram shown in Figure 4c. Hence, the intense peak of PbI_2_ in the diffractogram is a consequence of preferential orientation and not only abundance. In contrast, the ring at *q* = 1 Å^–1^ shows that FAPbI_3_ crystallites are mostly randomly oriented, although a certain degree of preferential orientation is present, as indicated by the higher intensity at about −45, 0, and 45° with respect to *q*_z_. Passivation with diluted BTaBr does not give rise to additional features compared to the reference film (Figure S17, Supporting Information). Angle-resolved GIWAXS allows us to characterize the film at different depths, as the penetration of the X-rays in the film is dependent on the angle of incidence. We performed such a measurement on FAPbI_3_ passivated with concentrated BTaBr. AR-GIWAXS with 0.1° incidence angle (probing the very top surface of the film) displays a Bragg spot at *q*_*z*_ = 0.45 Å^–1^ and a less intense one at 0.35 Å^–1^ (Figure 5b). These peaks likely correspond to I- and Br-rich 2D perovskites, respectively. It is unclear why we can discern both I- and Br-rich 2D perovskites via GIWAXS, whereas both XRD and PL hinted at the presence of only (BTa)_2_PbBr_4_. A possible explanation could be the presence of a more complex heterostructure, described as FAPbI_3_/(BTa)_2_PbI_4_/(BTa)_2_PbBr_4_. In such a case, the I-based 2D interlayer would likely not be visible in the PL because of efficient energy transfer to 3D but visible via GIWAXS. The main spot at 0.45 Å^–1^ resembles a half-ring, indicating that the 2D crystals possess a certain degree of disorder with random orientation along the *q_z_* plane. By increasing the incidence angle (until 0.5°, at which the whole film thickness is probed), the abovementioned PbI_2_ and FAPbI_3_ peaks appear as in the reference film (Figure 5c). This is in line with the presence of a superficial 2D perovskite layer on top of FAPbI_3_.

### Interlayer Characterization

We further characterized the heterostructure via XPS. First, we quantified the atomic concentrations of the elements composing the perovskite layer. As displayed in Table 2, the concentration of carbon (C) and nitrogen (N) at the surface increases with increasing BTaBr concentration. This indicates the presence of passivating molecules on the perovskite surface. Similarly, the bromine (Br) concentration increases from 1.2 to 5.7 at %, indicating a Br-richer surface, ideally because of the replacement of iodine(I) from (BTa)_2_PbI_4_ and conversion to (BTa)_2_PbBr_4_, as described above. As expected, the iodine concentration decreases with increasing passivation.

**Table 2:** Atomic Concentrations Obtained by XPS for FAPbI_3_ without and with 2D Interlayer Passivation

atomic concentration	Pb	N	C	I	Cl	Br
FAPbI_3_	9.6 ± 0.2	20.4 ± 0.9	18.5 ± 1.1	48.5 ± 0.8	1.7 ± 0.3	1.2 ± 0.1
FAPbI_3_ + BTaBr 1 mg mL^–1^	5.1 ± 0.1	23.8 ± 0.8	40.8 ± 1.3	24.5 ± 0.6	5.0 ± 0.5	0.7 ± 0.1
FAPbI_3_ + BTaBr 10 mg mL^–1^	1.6 ± 0.0	27.8 ± 0.4	57.0 ± 0.6	6.0 ± 0.1	1.7 ± 0.0	5.7 ± 0.1

**Figure 6:**
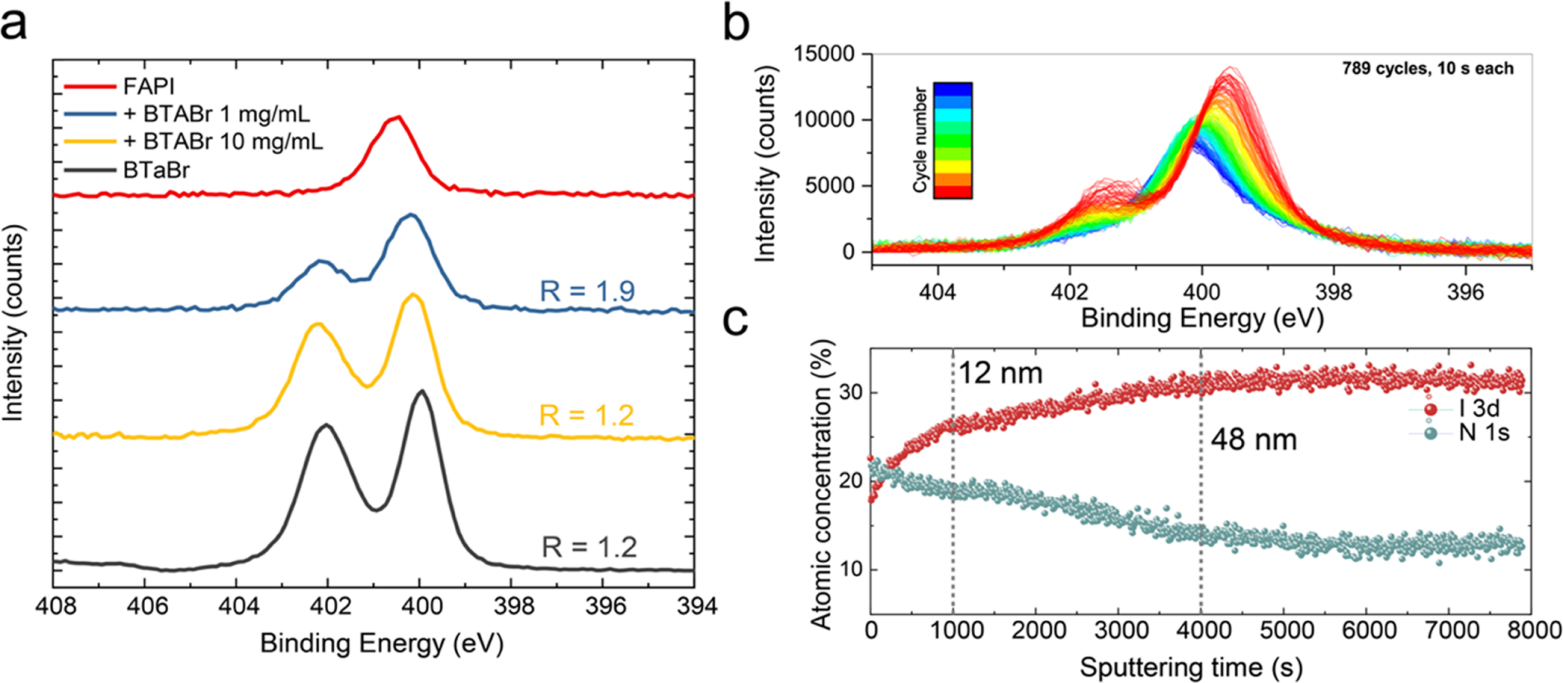
(a) N 1s XPS spectra for nonpassivated and passivated FAPbI_3_ films and for pure BTaBr. (b) Depth-profile N 1s XPS spectra for FAPbI_3_ + BTaBr 1 mg mL^–1^. (c) Atomic concentration variation for the same film as in panel (b).

By analyzing the N 1s spectrum and its depth profile, obtained via argon ion sputtering at low rates to avoid loss of features in the spectra (see the Experimental Section for more details), we gained more information on the formation of a 2D perovskite layer on top of the 3D one. First, nonpassivated FAPbI_3_ shows a single peak slightly above 400 eV for N 1s (Figure 6a). Then, once BTaBr is spin-coated onto the 3D layer, the films display a double peak in the same region. Here, the peak at ∼400 eV contains the contributions from both FAPbI_3_ and BTaBr, whereas the higher energy peak at ∼402 eV is only related to BTaBr. In pure BTaBr, the ratio between the low- and high-energy peaks is equal to 1.2, whereas it goes from 1.9 to 1.2 in the case of BTaBr 1 and 10 mg mL^–1^ deposited onto FAPbI_3_, respectively. These results indicate that BTaBr is present in both cases on top of the 3D perovskite but in different amounts. It seems that the XPS signal in the case of BTaBr 10 mg mL^–1^ originates prevalently from the 2D interlayer, whereas for the diluted BTaBr salt, there is a significant contribution from FAPbI_3_ as well. XPS is a surface-sensitive technique and we therefore speculate that after spin coating diluted BTaBr onto FAPbI_3_, the passivation layer might be either not closed or in the order of a few nm in thickness, thin enough to pick up signals from the underlying perovskite layer. On the contrary, when passivating with concentrated BTaBr, mostly the 2D layer is probed, indicating the formation of a much thicker interlayer that would negatively affect charge transport, as is indeed observed in the FF loss in the solar cell performance.

The depth profile gives additional valuable information on such an interlayer. After passivation with BTaBr 1 mg mL^–1^, the high-energy peak related to BTaBr decreases with increasing sputtering time, in line with a gradual removal of the passivation layer (Figure 6b). The low-energy peak, related to perovskite and BTaBr, shifts to higher binding energy over time and becomes equivalent to the one of pure FAPbI_3_. Again, this seems to indicate the presence of a rather thin film of BTa-based 2D perovskite or only sparse 2D crystallites on the surface, which would corroborate what was observed in SEM images.

By analyzing the atomic concentration variation of the sample with 1 mg mL^–1^ passivation, we found that the concentrations of iodine increase and those of nitrogen decrease over sputtering time (Figure 6c). In comparison, the concentrations of I and N are stable in the reference film (Figure S18, Supporting Information). The atomic concentration of nitrogen seems to follow three regimes, where first it decreases sharply, then linearly, and finally, it stabilizes and remains constant. In the first regime (till ∼1000 s sputtering time), the concentration of BTaBr molecules decreases fast, indicating direct removal of the interlayer. In the second regime (till ∼4000 s), the concentration of nitrogen decreases more gradually; this might indicate that BTaBr molecules penetrate the perovskite bulk, possibly forming (BTa)_2_FAPb_2_I_7_, and such a removal is slower and happens together with the sputtering of the 3D perovskite layer. Finally, in the final regime, the concentration becomes constant, which indicates the presence of a conventional FAPbI_3_ layer. The N 1s spectra in these three regimes are displayed in Figure S19 (Supporting Information). We additionally measured the thickness of the sputtered area after ∼790 cycles of 10 s via a profilometer and found the thickness to be 93 nm. The etching rate on the perovskite was thus calculated as 0.012 nm s^–1^. With this information, we calculated that the 2D interlayer is in the order of 12 nm, whereas the penetration of the BTaBr molecules goes as deep as ∼48 nm.

For FAPbI_3_ passivated with BTaBr 10 mg mL^–1^, the two N 1s peaks are still visible even with rougher sputtering conditions and indicate the presence of a thicker 2D interlayer (Figure S20, Supporting Information). The same rougher sputtering conditions for FAPbI_3_ + BTaBr 1 mg mL^–1^ removed all BTaBr molecules after only one cycle. This confirms the presence of a much thicker 2D interlayer when passivating with concentrated BTaBr.

## Conclusions

In conclusion, we introduced a novel benzotriazole-based cation to form 2D and quasi-2D perovskites and showed that it is beneficial to passivate the surface of FAPbI_3_ perovskites with such a cation to enhance solar cells parameters. A solar cell based on FAPbI_3_ passivated with an optimal amount of BTaBr reached almost 22% PCE. Mainly, *V*_OC_ increase is behind the enhancement of photovoltaic performance. By using QFLS and SEM-CL, we demonstrated that BTaBr indeed passivates FAPbI_3_ and reduces nonradiative recombination at the perovskite/HTL interface. With a variety of characterization methods, we provided insights into the formation of a 2D interlayer. We showed that 2D perovskites, either iodide- or bromide-based, are formed even with low amounts of spacers, but the nature of the resulting layer can vary. Low concentration of the 2D spacer leads to the formation of iodide-based 2D and quasi-2D perovskites. It is likely that such a low concentration does not lead to the formation of a closed 2D interlayer but rather results in sparse crystallites of 2D perovskites being formed on the surface of the 3D perovskite, which are still beneficial to reduce nonradiative recombination and enhance device performance. With increasing concentration of BTaBr, a thicker 2D, bromide-based perovskite layer is formed, which is detrimental for charge extraction. With this study, further knowledge on the formation of 3D/2D heterostructures is developed. Furthermore, the easy-to-functionalize nature of benzotriazole cations suggests that such spacers could pave the way to functionalized 2D interlayers that could push the efficiency and stability of perovskite solar cells even further.

## Supporting Information Available

Additional experimental details and methods; ^1^H NMR and ^13^C NMR spectra of synthesized compounds; single-crystal data of the 2D perovskites, including UV–vis, PL, XPS spectra, X-ray diffractograms, SEM and SEM-Cl images, and device statistics (PDF)
