# Supplementary material for: 3D Perovskite Passivation with a Benzotriazole-Based 2D Interlayer for High-Efficiency Solar Cells
Source: ACS Appl Energy Mater. 2023 Mar 27;6(7):3933–43. doi: 10.1021/acsaem.3c00101 (PMC10091350; doi:10.1021/acsaem.3c00101)
Supplement: Supplementary file 1 — ae3c00101_si_001.pdf [file ae3c00101_si_001.pdf]

Supporting Information

3D perovskite passivation with a benzotriazole-based 2D interlayer for high efficiency solar cells

Alessandro Caiazzo,[1] *Arthur Maufort,*[2] *Bas T. van Gorkom,*[1] *Willemijn H. M. Remmerswaal,*[1] *Jordi Ferrer Orri,*[3,4,5] *Junyu Li,*[1] *Junke Wang,*[1] *Wouter T. M. Van Gompel,*[2] *Kristof Van Hecke,*[6] *Gunnar Kusch,*[4] *R. A. Oliver*[4], *Caterina Ducati,*[4] *Laurence Lutsen,*[2] *Martijn M. Wienk,*[1] *Samuel D. Stranks,*[3,5] *Dirk Vanderzande,*[2] *René A. J. Janssen*[1,7*]

[1] Molecular Materials and Nanosystems and Institute of Complex Molecular Systems Eindhoven University of Technology, P.O. Box 513, 5600 MB Eindhoven, The Netherlands, E-mail: r.a.j.janssen@tue.nl.

[2] Institute for Materials Research (IMO-IMOMEC), Hybrid Materials Design, Hasselt University, Martelarenlaan 42, B-3500 Hasselt, Belgium.

[3] Cavendish Laboratory, University of Cambridge, Cambridge CB3 0HE, United Kingdom

[4] Department of Materials Science and Metallurgy, University of Cambridge, Cambridge CB3 0FS, United Kingdom.

[5] Department of Chemical Engineering and Biotechnology, University of Cambridge, Cambridge CB3 0HE, United Kingdom.

[6] XStruct, Department of Chemistry, Ghent University, Krijgslaan 281-S3, B-9000, Ghent, Belgium.

[7] Dutch Institute for Fundamental Energy Research, De Zaale 20, 5612 AJ Eindhoven, The Netherlands.

*Corresponding author: r.a.j.janssen@tue.nl

Experimental

1. Synthesis of benzotriazole derivatives

Materials: All commercial chemicals and solvents were used without additional purification steps unless stated otherwise. 1,2-diamino-4,5-difluorobenzene (98%), N-Boc-ethanolamine, triphenylphosphine, and HBr (48% in water) were purchased from Fluorochem. Triphenylphosphine was recrystallized from methanol before use. 1H-benzotriazole (99%) was purchased from Alfa Aesar. Di-2-methoxyethyl azodicarboxylate (DMEAD) (≥90%) and sodium nitrite were purchased from Sigma-Aldrich. HI (57% in water, distilled, unstabilized) and tri-n-butyl phosphate (>99%) were purchased from Acros Organics. All solvents were purchased from Fisher Scientific.

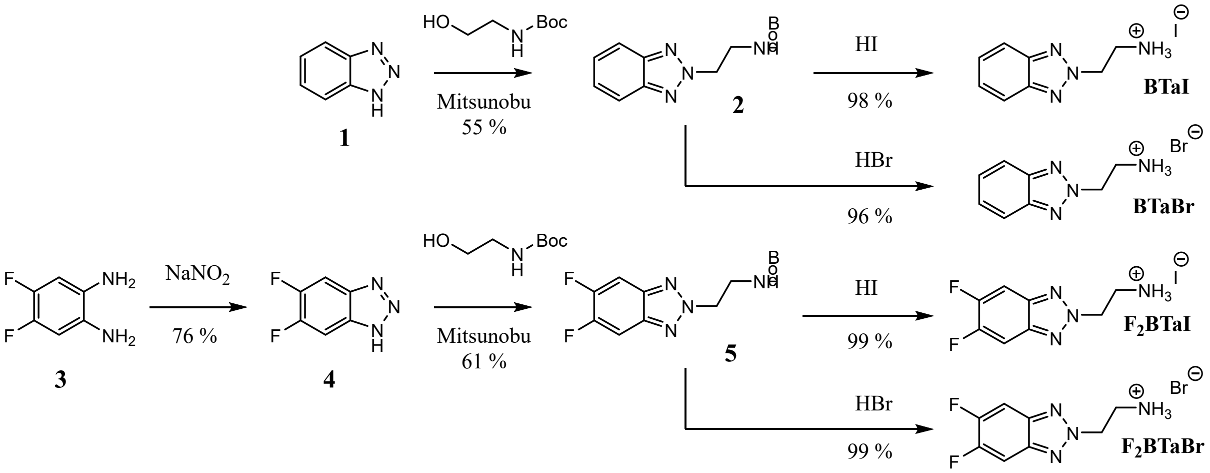

Scheme S1. Synthesis Route Towards Organic Ammonium Halides.

2-(2*H*-benzo[*d*][1,2,3]triazol-2-yl)ethylamine, Boc protected (2)

Boc-ethanolamine (4.5 g, 27.7 mmol) was weighed in a flame-dried 3-neck round-bottom flask; then **1** (3.0 g, 25.2 mmol), triphenylphosphine (7.3 g, 27.7 mmol), and dry diethyl ether (60 mL) were added. The dispersion was brought under Ar atmosphere and cooled to 0 °C. DMEAD (6.5 g, 27.7 mmol) was dissolved in 30 mL dry diethyl ether and was added dropwise to the reaction mixture. After DMEAD had been added completely, the reaction mixture was slowly brought back to ambient temperature. After 18 h the reaction mixture was extracted with water,

and the water fractions were combined and extracted with diethyl ether. The combined ether fractions were dried and filtered, and the solvent was evaporated under reduced pressure. The resulting crude product was purified by column chromatography with gradient elution from DCM to DCM/EtOAc 9:1 to obtain **2** as a white solid (3.63 g, 55% yield).). ^{1}H NMR (400 MHz, Chloroform-*d*) *δ* 7.89 – 7.81 (m, 2H), 7.41 – 7.35 (m, 2H), 5.11 (s, 1H), 4.86 – 4.78 (m, 2H), 3.85 (q, J = 5.8 Hz, 2H), 1.41 (s, 9H). ^{13}C NMR (101 MHz, Chloroform-*d*) *δ* 155.79, 144.51, 126.57, 118.10, 79.89, 56.49, 40.11, 28.41. GC-MS: *m*/*z* = 262.

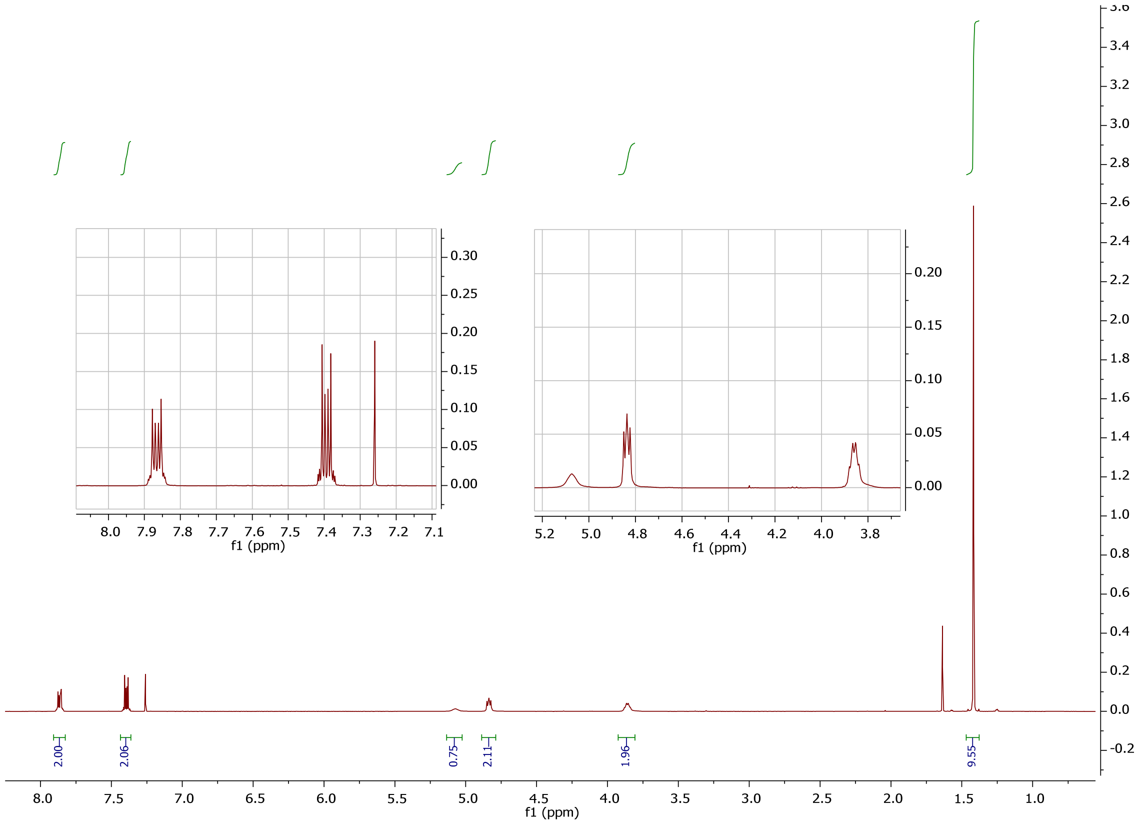

^{1}H NMR spectrum (chloroform at 7.26 ppm; water at 1.63 ppm)

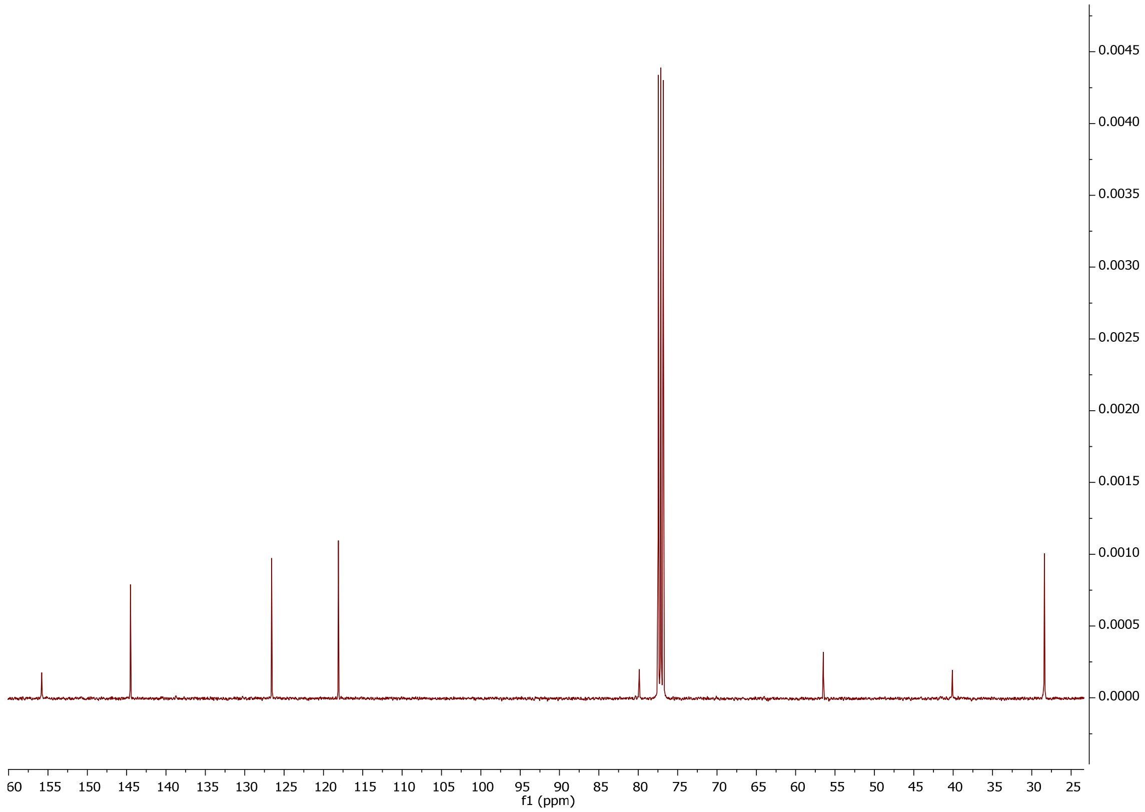

^{13}C NMR spectrum (chloroform at 77 ppm)

Boc-protected 2-(1*H*-benzo[*d*][1,2,3]triazol-1-yl)ethylamine (asymmetrically alkylated benzotriazole) was also obtained as a side product. ^{1}H NMR (400 MHz, Chloroform-*d*) δ 7.98 (d, J = 8.4 Hz, 1H), 7.53 (d, J = 8.4 Hz, 1H), 7.45 (ddd, J = 8.2, 6.8, 1.0 Hz, 1H), 7.33 (ddd, J = 8.0, 6.7, 1.1 Hz, 1H), 4.98 (t, J = 6.1 Hz, 1H), 4.74 (t, J = 5.8 Hz, 2H), 3.70 (q, J = 5.9 Hz, 2H), 1.38 (s, 9H).

2-(2*H*-benzo[*d*][1,2,3]triazol-2-yl)ethylammonium iodide (BTaI)

HI (57%, unstabilized) was extracted three times with a 9:1 mixture of chloroform and tributyl phosphate to remove impurities. 1.11 mL (8.39 mmol) of this freshly extracted HI was then added with a micropipette to a solution of **2** (1.010 g, 3.81 mmol) in dioxane (25 mL), upon which the color of the mixture changed from colorless to yellow. The reaction mixture was flushed with Ar and was left to react at ambient temperature in the dark for 15 h. The mixture

was then concentrated under reduced pressure, and the resulting solid was sonicated with diethyl ether, filtered, and washed several times with diethyl ether. **BTaI** was obtained as a white solid (1.095 g, 98% yield) and was dried under high vacuum. ^{1}H NMR (400 MHz, DMSO-d_6) δ 8.04 – 7.87 (m, 5H), 7.48 – 7.40 (m, 2H), 5.02 – 4.94 (m, 2H), 3.56 – 3.48 (m, 2H). ^{13}C NMR (101 MHz, DMSO-d_6) δ 144.49, 127.26, 118.49, 53.85, 38.99.

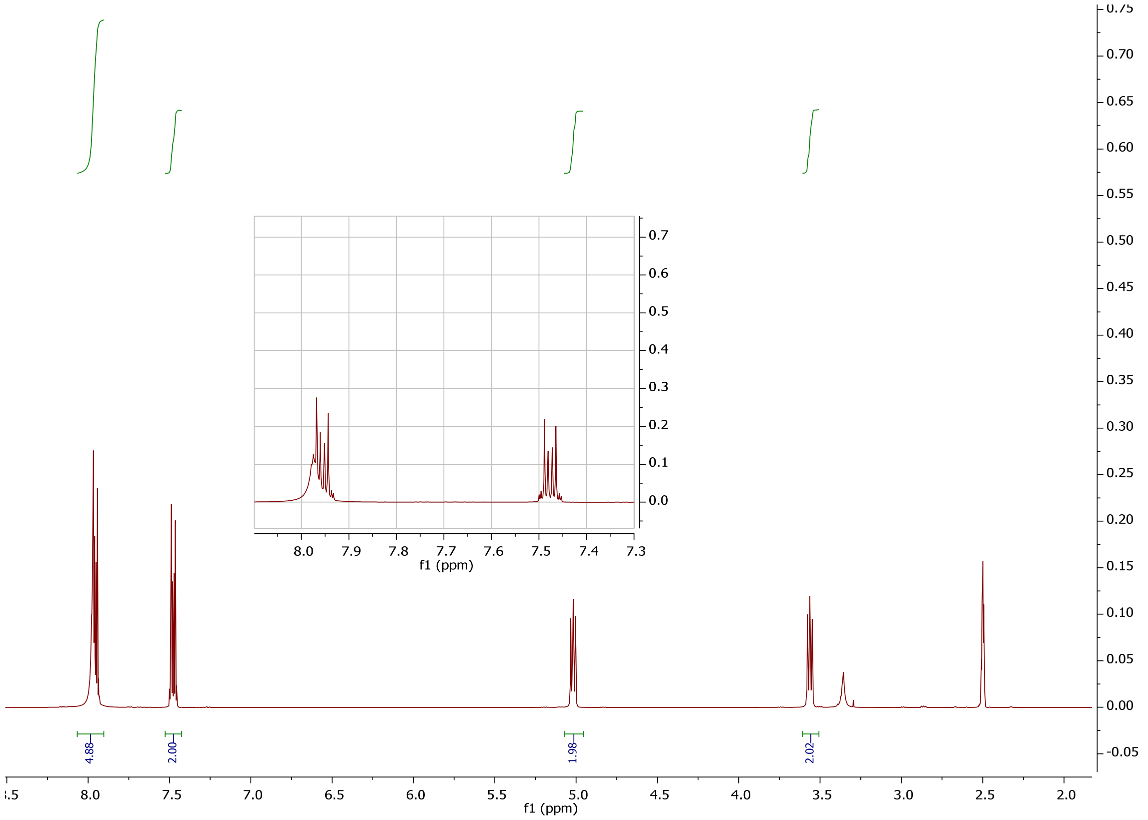

^{1}H NMR spectrum (DMSO at 2.50 ppm; water at 3.36 ppm)

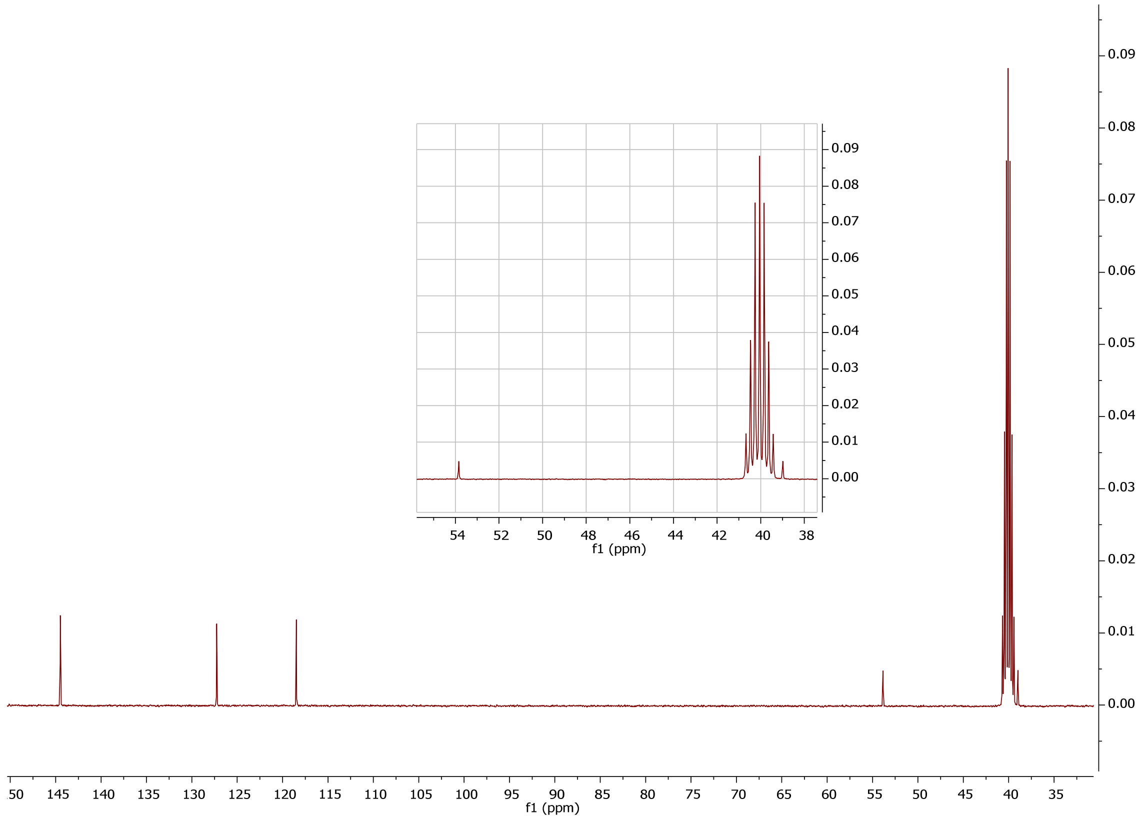

^{13}C NMR spectrum (DMSO at 40 ppm)

2-(2*H*-benzo[*d*][1,2,3]triazol-2-yl)ethylammonium bromide (BTaBr)

2 (1.000 g, 3.81 mmol) was dissolved in dioxane (25 mL), after which HBr (48%, 949 μL, 8.39 mmol) was added with a micropipette. The mixture was flushed with Ar and was left to react at ambient temperature for 17 h, during which a precipitate formed. The mixture was then concentrated under reduced pressure, sonicated in diethyl ether, filtered, and washed several times with diethyl ether to yield **BTaBr** as a white powder (0.890 g, 96% yield), which was dried under high vacuum. ^{1}H NMR (400 MHz, DMSO-d_6) δ 7.97 (s, 3H), 7.95 – 7.89 (m, 2H), 7.48 – 7.41 (m, 2H), 5.03 – 4.94 (m, 2H), 3.56 – 3.48 (m, 2H). ^{13}C NMR (101 MHz, DMSO-d_6) δ 144.49, 127.23, 118.48, 53.72, 38.88.

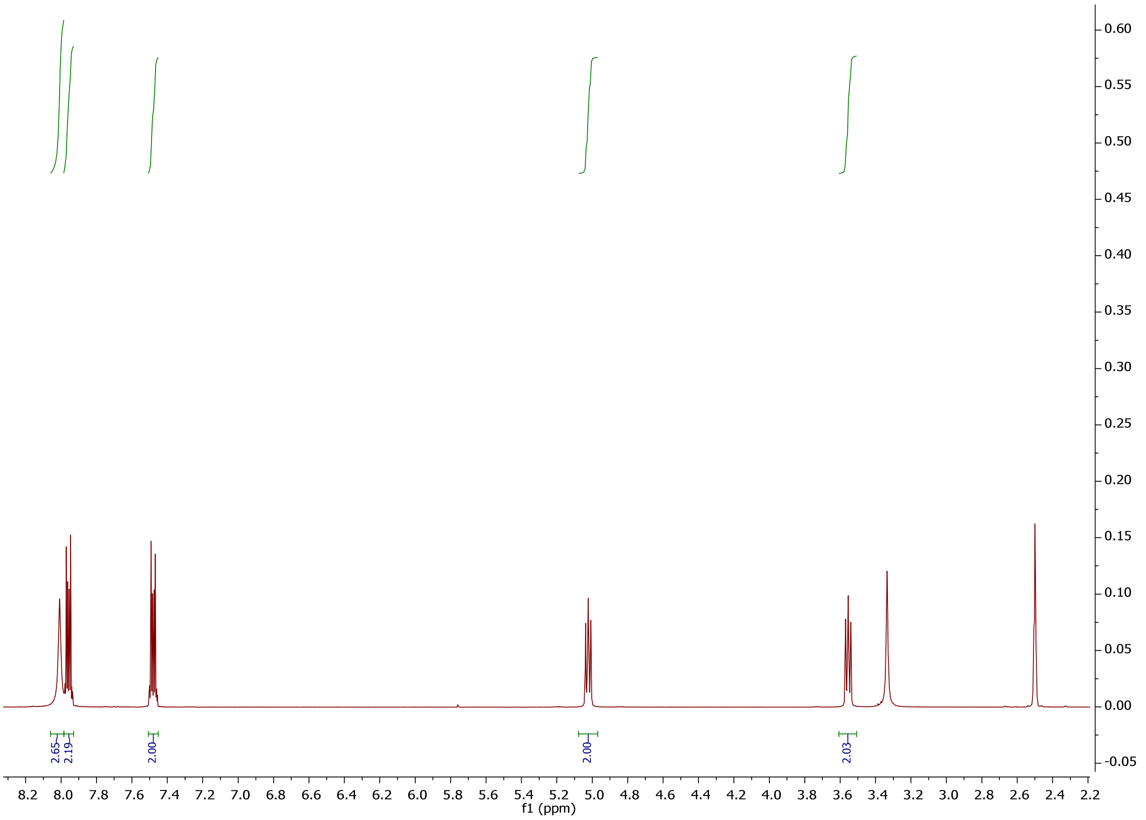

^{1}H NMR spectrum (DMSO at 2.50 ppm; water at 3.33 ppm)

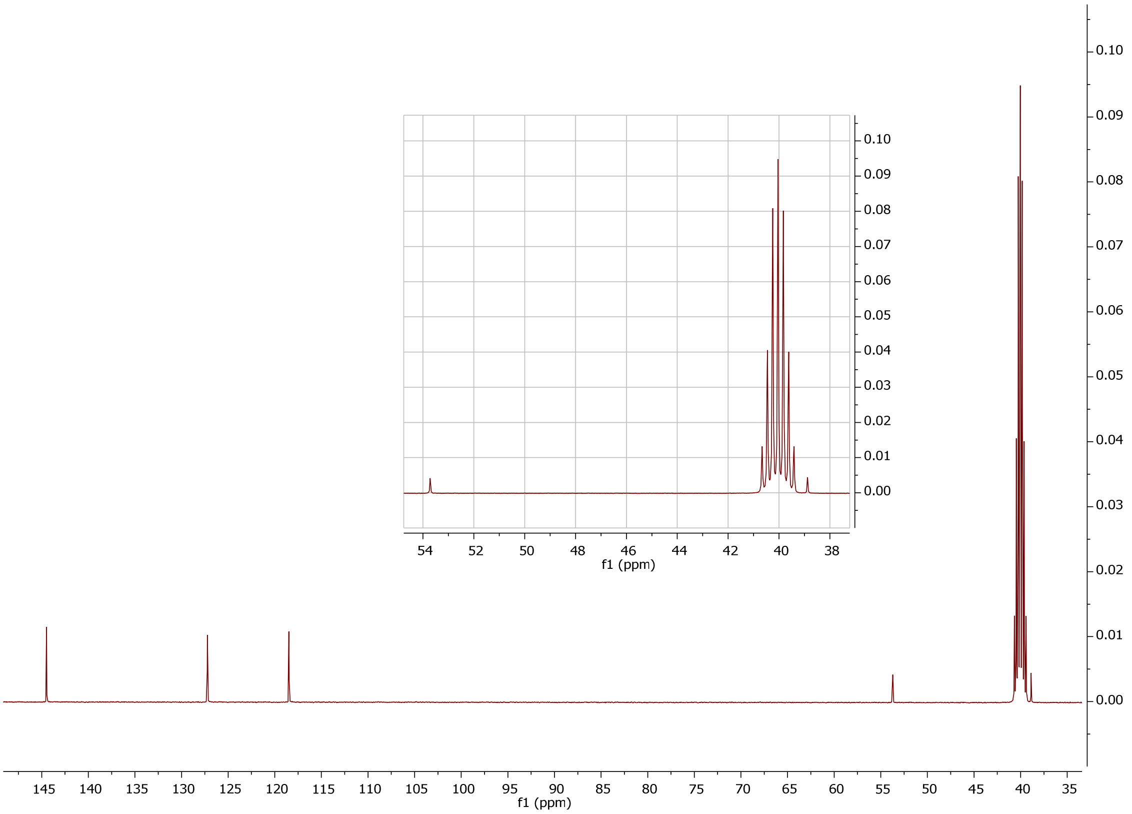

^{13}C NMR spectrum (DMSO at 40 ppm)

5,6-difluoro-1*H*-benzo[*d*][1,2,3]triazole (4)

3 (2.992 g, 20.8 mmol) was dispersed in water (100 mL) and acetic acid (2.4 mL, 42.0 mmol) and was heated under reflux. The mixture was then filtered over a glass filter; the filtrate was slowly cooled down to ambient temperature and was subsequently put in the fridge (5 °C). Meanwhile, sodium nitrite (1.674 g, 24.3 mmol) was dissolved in water (40 mL), and the resulting solution was also put in the fridge. After both solutions had been at 5 °C for 1 h, the sodium nitrite solution was poured into the diamine solution at 5 °C, after which it was slowly brought back to ambient temperature. A precipitate formed, which was filtered off after 1 h. This solid was recrystallized from water and dried under high vacuum to yield **4** as an orange solid (2.45 g, 76% yield). ^{1}H NMR (400 MHz, DMSO-d_6) δ 15.97 (s, 1H), 8.10 – 7.97 (m, 2H). ^{13}C NMR (101 MHz, DMSO-d_6) δ 149.67 (dd, J = 245.6, 16.7 Hz), 134.99, 102.83. ^{19}F NMR (376 MHz, DMSO-d_6) δ −137.39. GC-MS: m/z = 155.

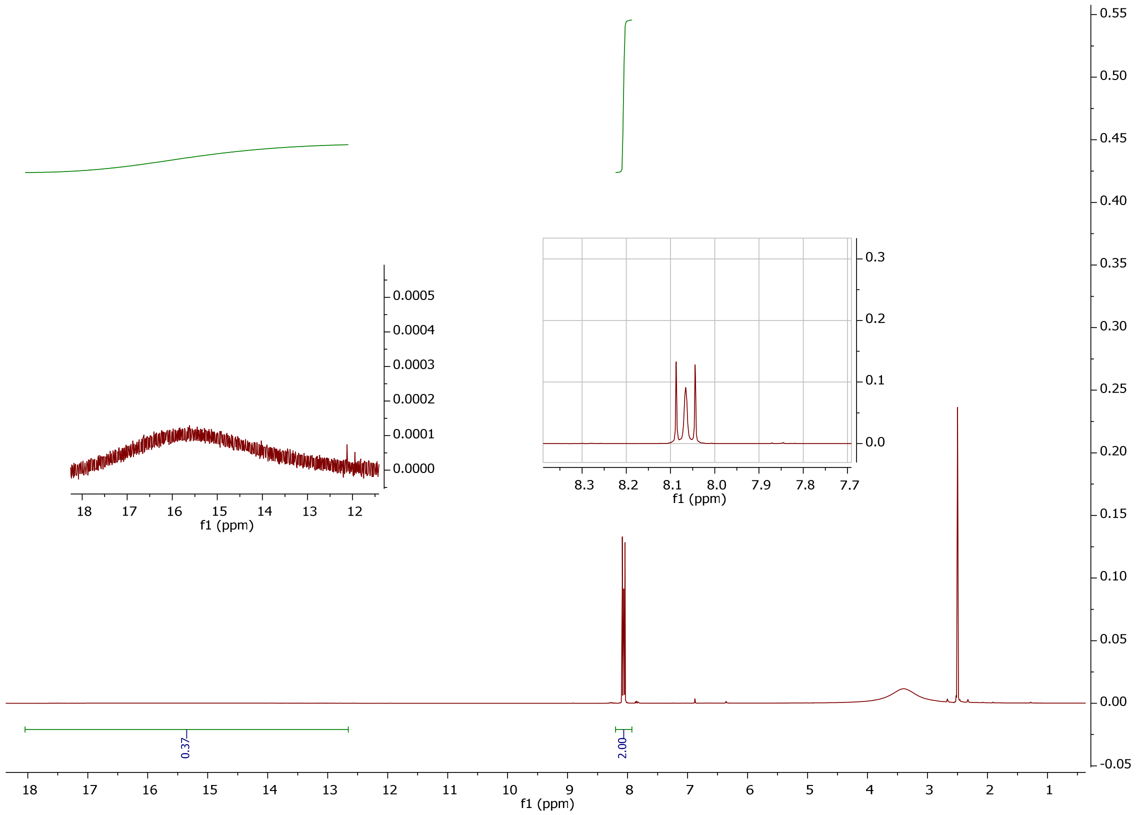

^{1}H NMR spectrum (DMSO at 2.50 ppm; water at 3.38 ppm)

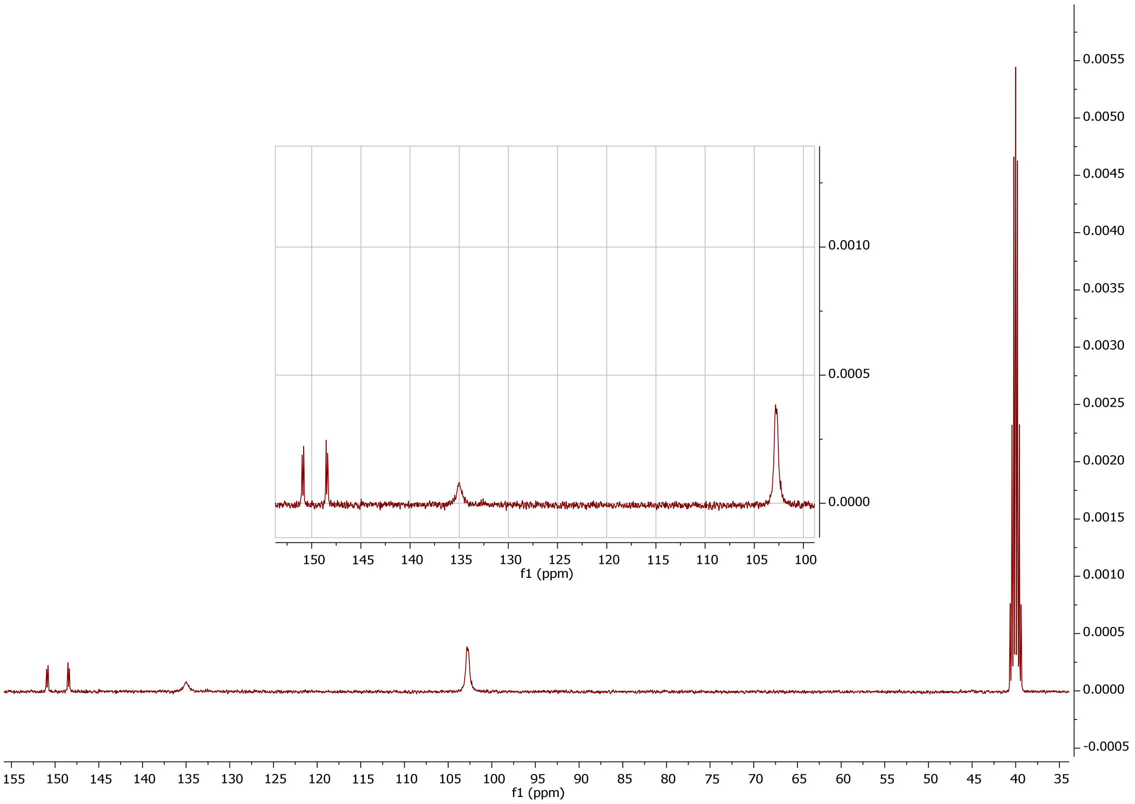

^{13}C NMR spectrum (DMSO at 40 ppm)

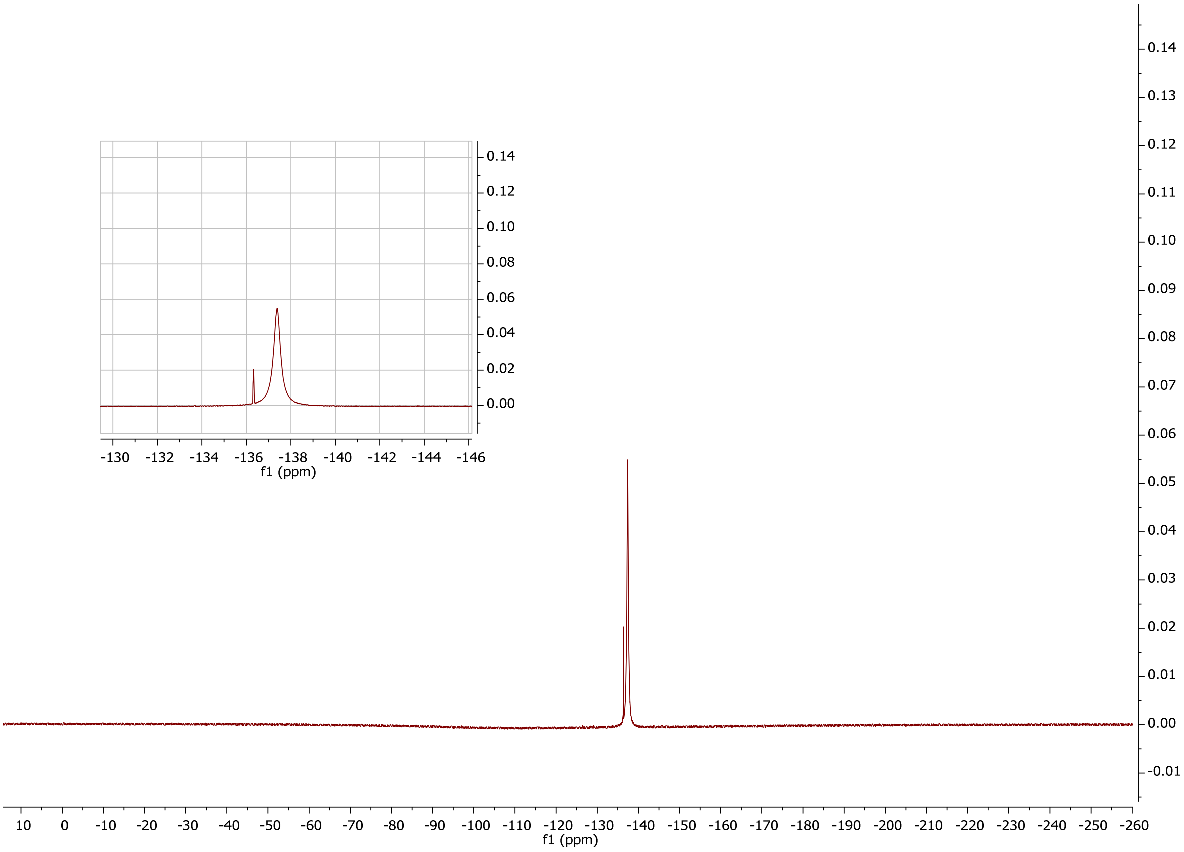

^{19}F NMR spectrum

2-(5,6-difluoro-2*H*-benzo[*d*][1,2,3]triazol-2-yl)ethylamine, Boc protected (5)

Boc-ethanolamine (1.203 g, 7.46 mmol) was weighed in a flame-dried 3-neck round-bottom flask, then **4** (1.034 g, 6.67 mmol) and triphenylphosphine (1.957 g, 7.46 mmol) were added. Dry diethyl ether (70 mL) was subsequently added, and the resulting dispersion was sonicated for 1 min to break down **3** to a fine powder. The dispersion was then brought under Ar atmosphere and cooled to 0 °C. DMEAD (1.747 g, 7.46 mmol) was dissolved in a small amount of dry diethyl ether and was added dropwise to the reaction mixture. After DMEAD had been added completely, the reaction mixture was slowly brought back to ambient temperature. The dispersion became a clear solution after about 10 minutes. After 18 h the reaction mixture was extracted with water, and the water fractions were combined and extracted with diethyl ether. The combined ether fractions were dried and filtered, and the solvent was evaporated under reduced pressure. The resulting crude product was purified by column chromatography with gradient elution from DCM/EtOAc 9:1 to 8:2 to obtain **5** as an off-white solid (1.220 g, 61% yield). Also Boc-protected 2-(5,6-difluoro-1H-benzo[d][1,2,3]triazol-1-yl)ethylamine (asymmetrically alkylated benzotriazole) was obtained as a side product (0.230 g, 12% yield). ^{1}H NMR (400 MHz, Chloroform-*d*) δ 7.58 (t, J = 8.4 Hz, 2H), 4.98 (s, 1H), 4.84 – 4.72 (m, 2H), 3.81 (q, J = 5.8 Hz, 2H), 1.40 (s, 9H). ^{13}C NMR (101 MHz, Chloroform-*d*) δ 155.74, 151.47 (dd, J = 252.4, 19.2 Hz), 140.23 (t, J = 5.9 Hz), 104.19 – 103.33 (m), 80.01, 56.64, 40.06, 28.38. ^{19}F NMR (376 MHz, Chloroform-*d*) δ −133.17 (t, J = 8.4 Hz). GC-MS: m/z = 298.

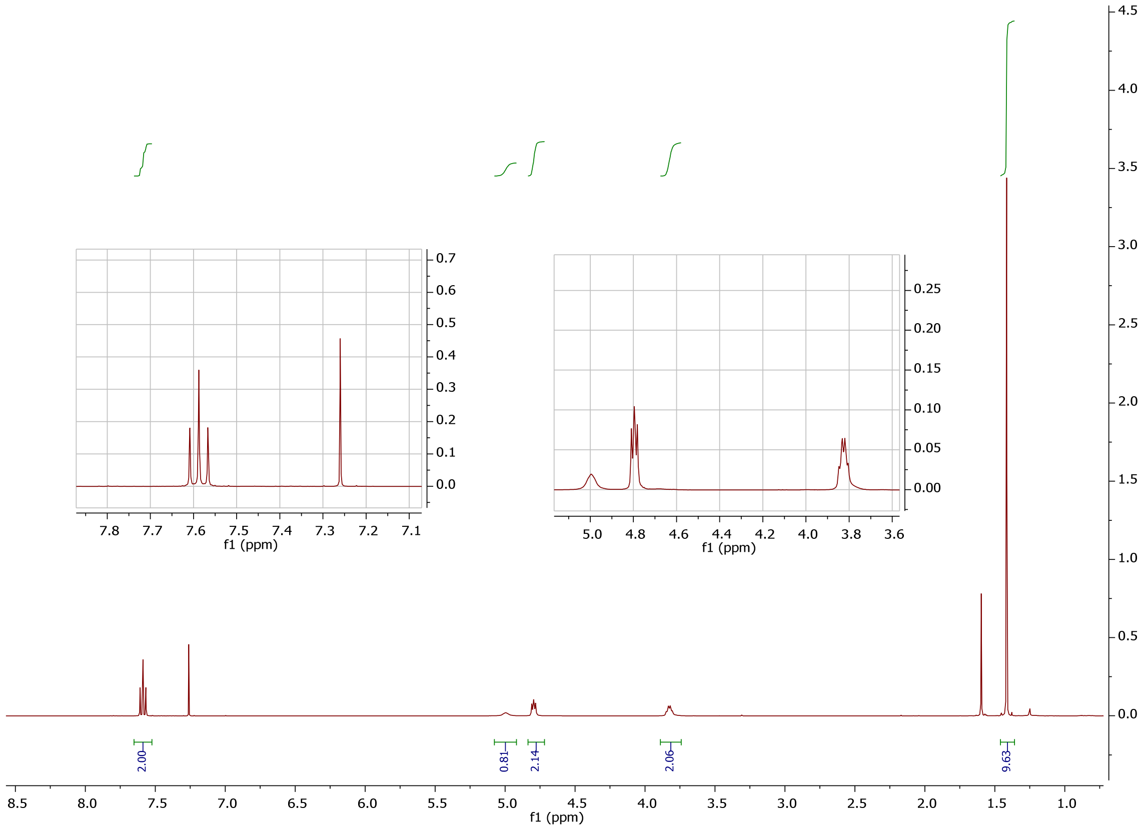

^{1}H NMR spectrum (chloroform at 7.26 ppm; water at 1.59 ppm)

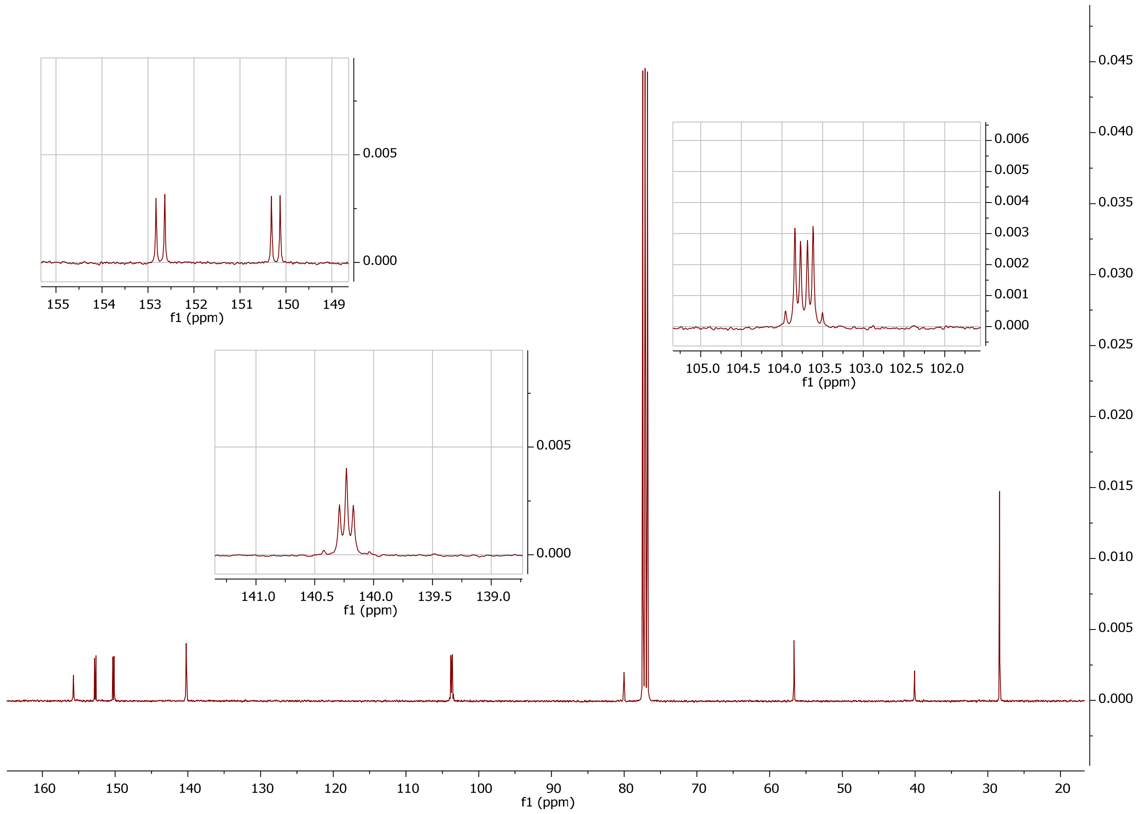

^{13}C NMR spectrum (chloroform at 77 ppm)

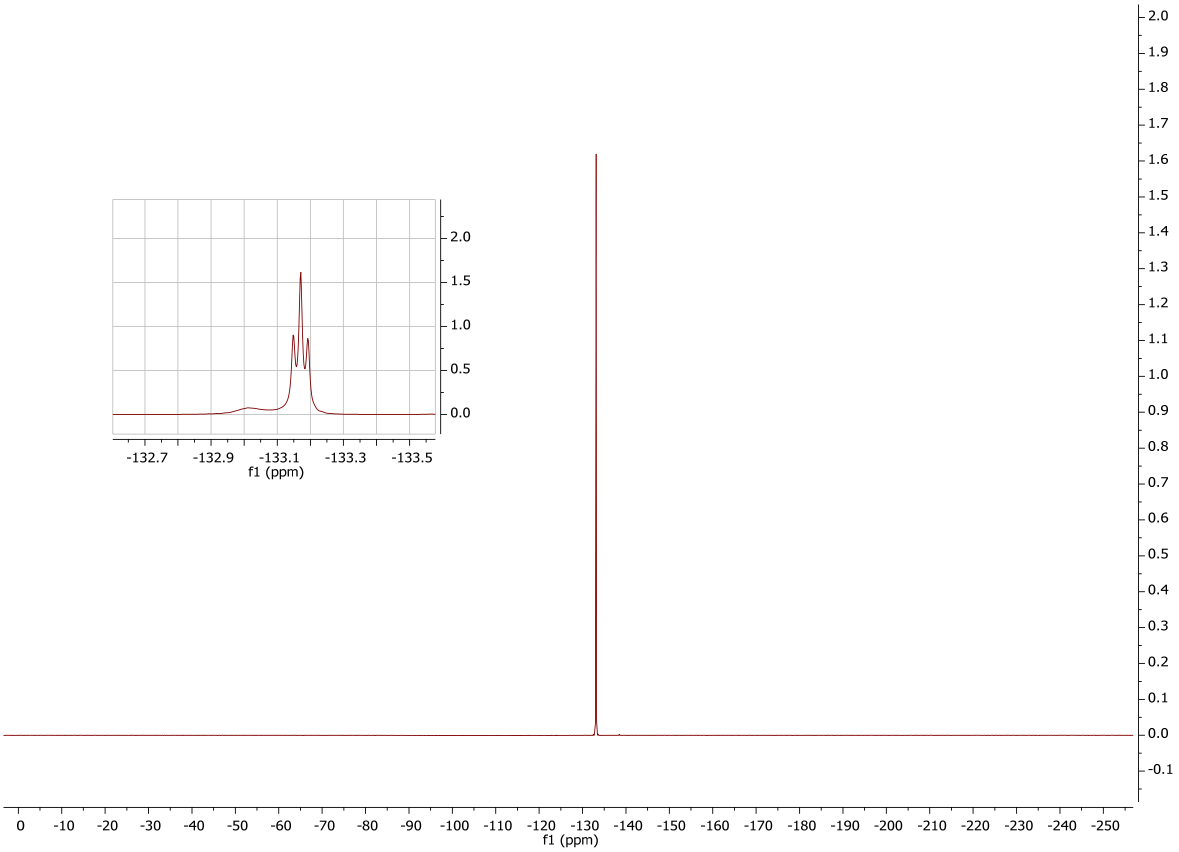

^{19}F NMR spectrum

2-(5,6-difluoro-2*H*-benzo[*d*][1,2,3]triazol-2-yl)ethylammonium iodide (F_2BTaI)

HI (57%, unstabilized) was extracted three times with a 9:1 mixture of chloroform and tributyl phosphate to remove impurities. 778 µL (5.90 mmol) of this freshly extracted HI was then added with a micropipette to a solution of **5** (0.797 g, 2.68 mmol) in dioxane (10 mL), upon which the color of the mixture changed from yellow to orange. The reaction mixture was flushed with Ar and was left to react at ambient temperature in the dark for 15 h. The mixture was then concentrated under reduced pressure, and the resulting solid was sonicated with diethyl ether, filtered, and washed several times with diethyl ether. **F_2BTaI** was obtained as a pale-yellow solid (0.863 g, 99% yield) and was dried under high vacuum. ^{1}H NMR (400 MHz, DMSO-d_6) δ 8.07 (t, J = 8.9 Hz, 2H), 7.91 (s, 3H), 5.01 – 4.90 (m, 2H), 3.53 – 3.45 (m, 2H).

^{13}C NMR (101 MHz, DMSO-d_6) δ 151.06 (dd, J = 249.2, 19.4 Hz), 140.52 (t, J = 6.2 Hz), 105.32 – 104.64 (m), 54.29, 39.10. ^{19}F NMR (376 MHz, DMSO-d_6) δ −134.36 (t, J = 8.9 Hz).

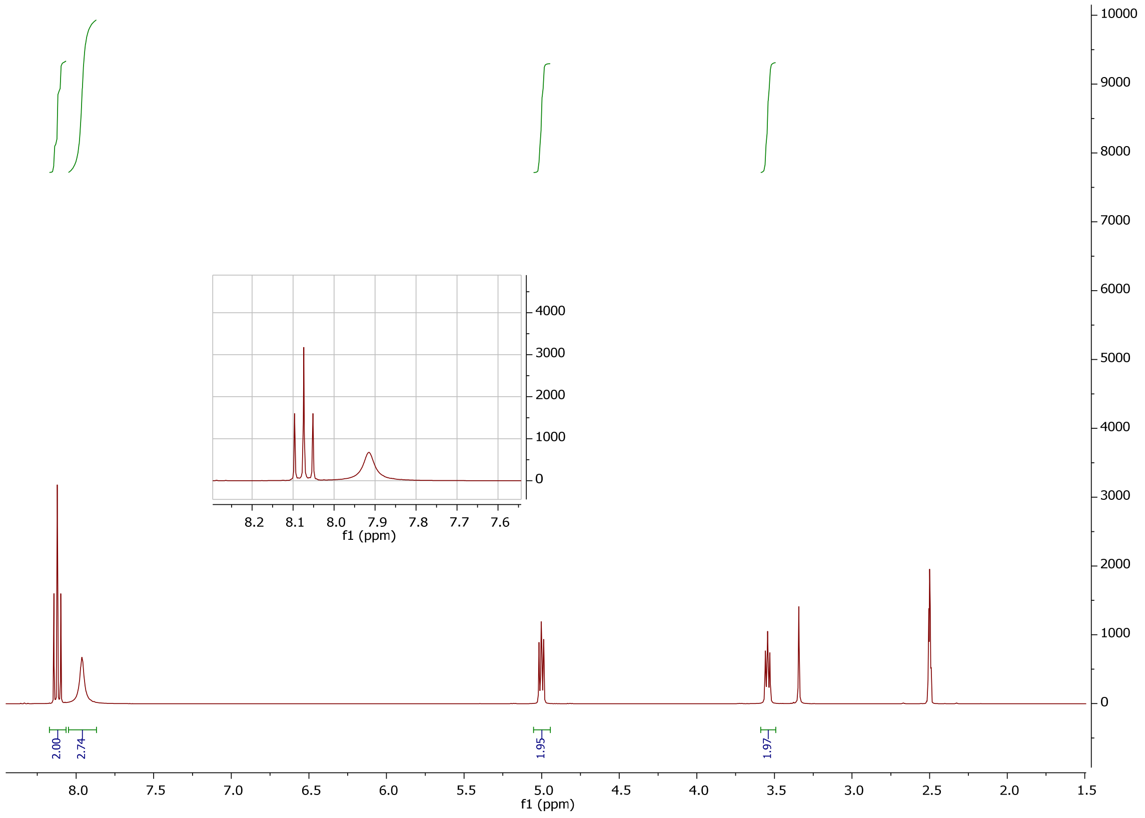

^{1}H NMR spectrum (DMSO at 2.50 ppm; water at 3.33 ppm)

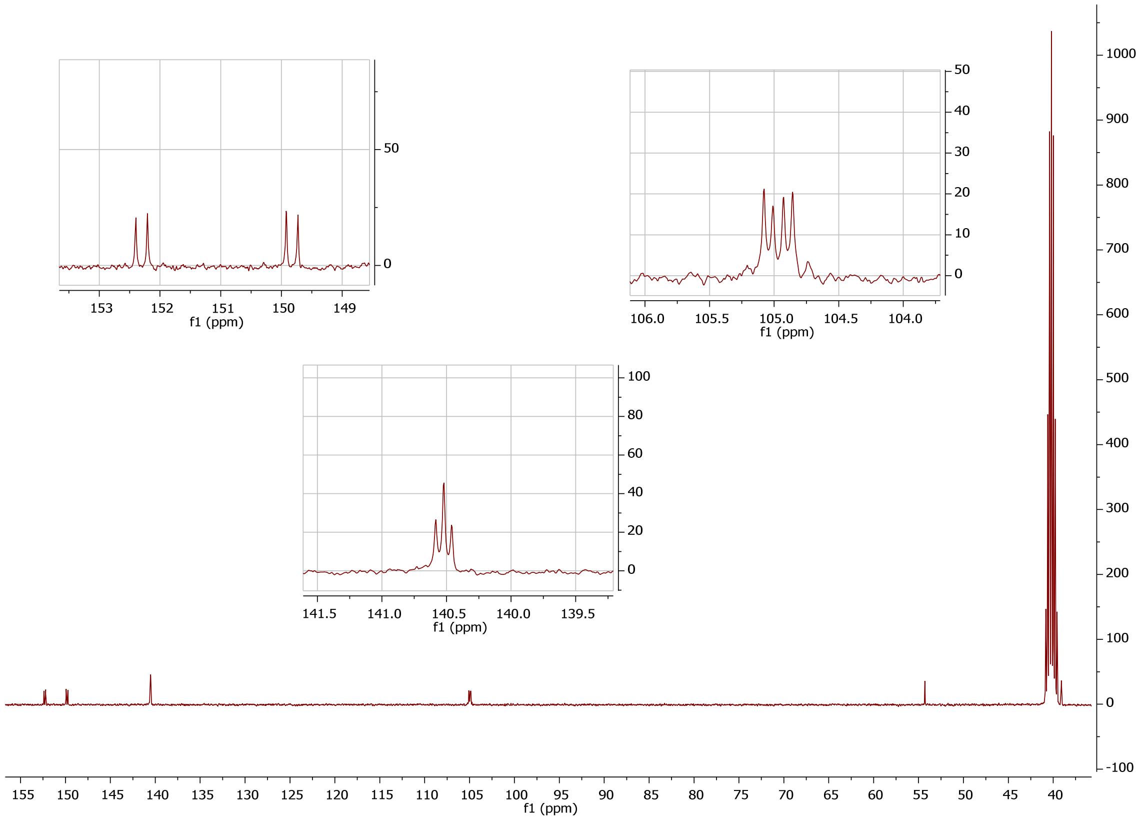

^{13}C NMR spectrum (DMSO at 40 ppm)

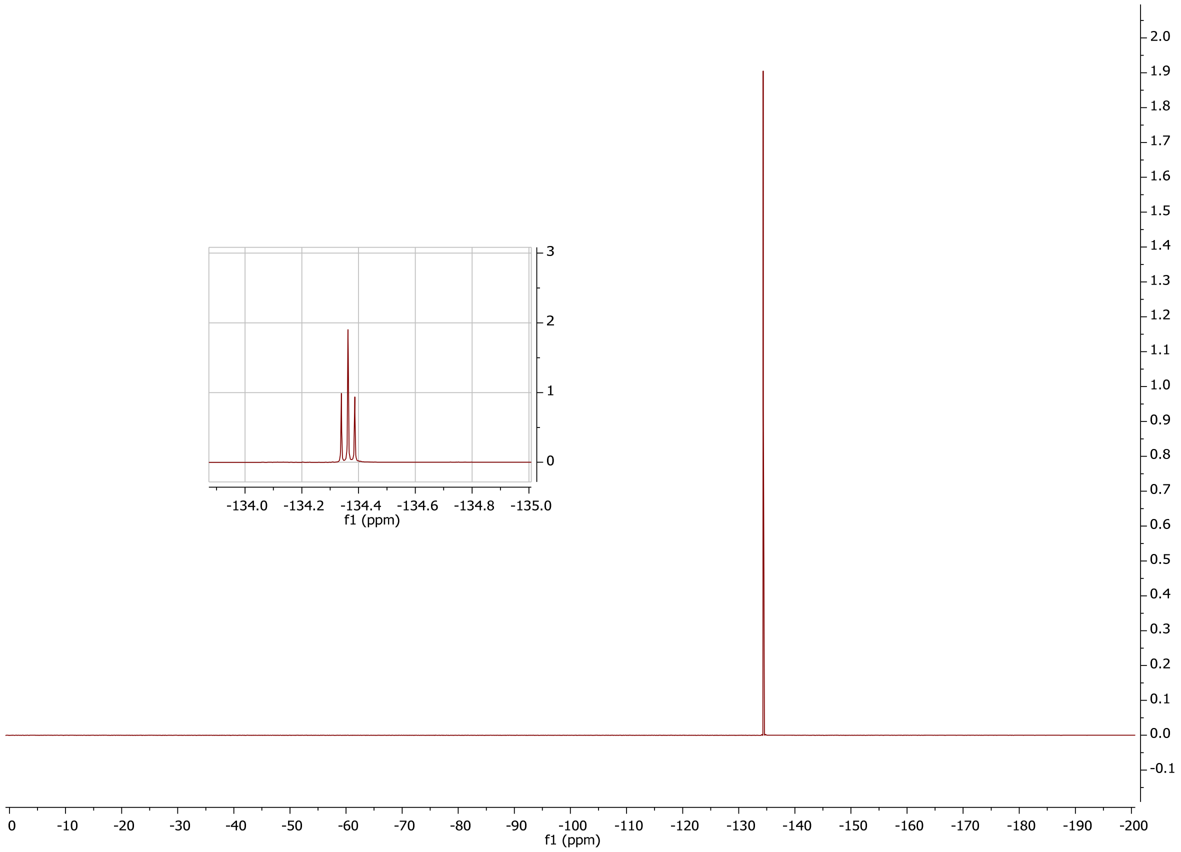

^{19}F NMR spectrum

2-(5,6-difluoro-2*H*-benzo[*d*][1,2,3]triazol-2-yl)ethylammonium bromide (F_2BTaBr)

5 (0.498 g, 1.67 mmol) was dissolved in dioxane (10 mL), after which HBr (48%, 417 µL, 3.69 mmol) was added with a micropipette. The mixture was flushed with Ar and was left to react at ambient temperature for 21 h, during which the colour changed from yellow to orange and a precipitate formed. The mixture was then concentrated under reduced pressure, sonicated in diethyl ether, filtered, and washed several times with diethyl ether to yield **F_2BTaBr** as an off-white powder (0.460 g, 99% yield), which was dried under high vacuum. ^{1}H NMR (400 MHz, DMSO-d_6) δ 8.10 (t, J = 8.9 Hz, 2H), 7.94 (s, 3H), 5.00 – 4.94 (m, 2H), 3.53 – 3.46 (m, 2H). ^{13}C NMR (101 MHz, DMSO-d_6) δ 150.90 (dd, J = 249.2, 19.3 Hz), 140.36 (t, J = 6.2 Hz), 105.15 – 104.41 (m), 54.00, 38.83. ^{19}F NMR (373 MHz, DMSO-d_6) δ −134.36 (t, J = 8.9 Hz).

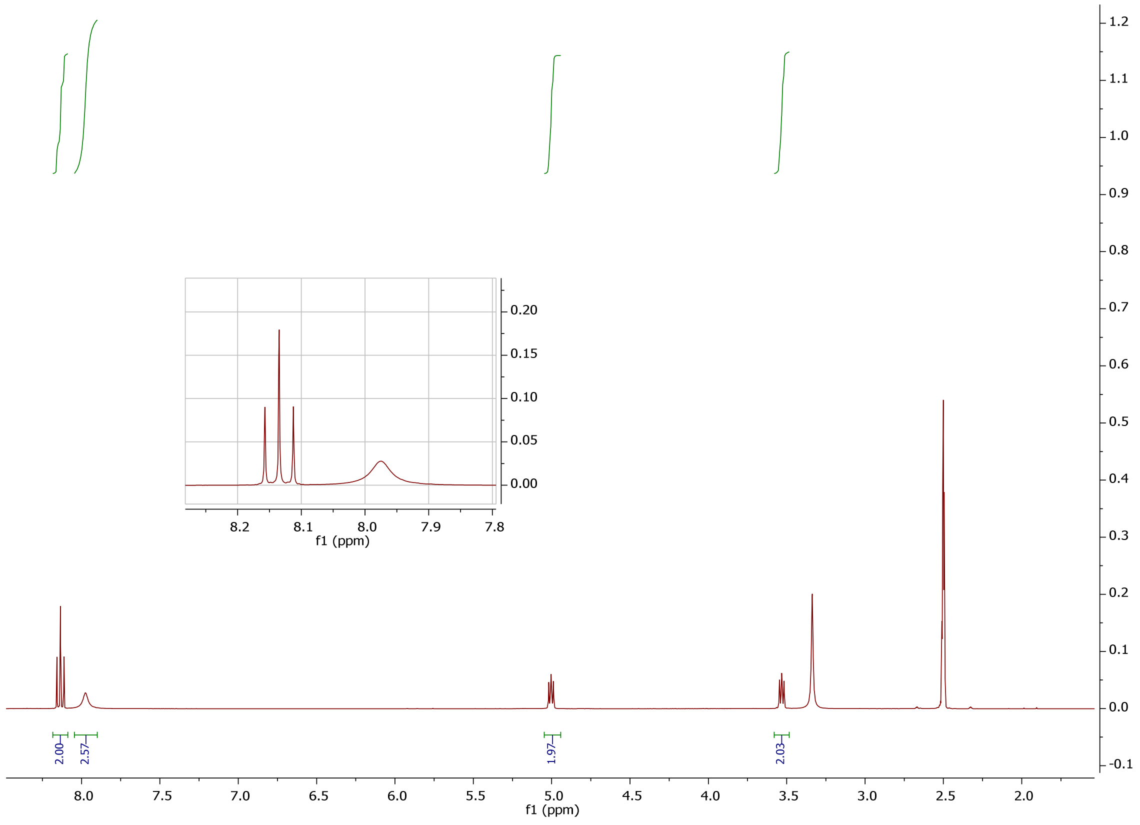

^{1}H NMR spectrum (DMSO at 2.50 ppm; water at 3.34 ppm)

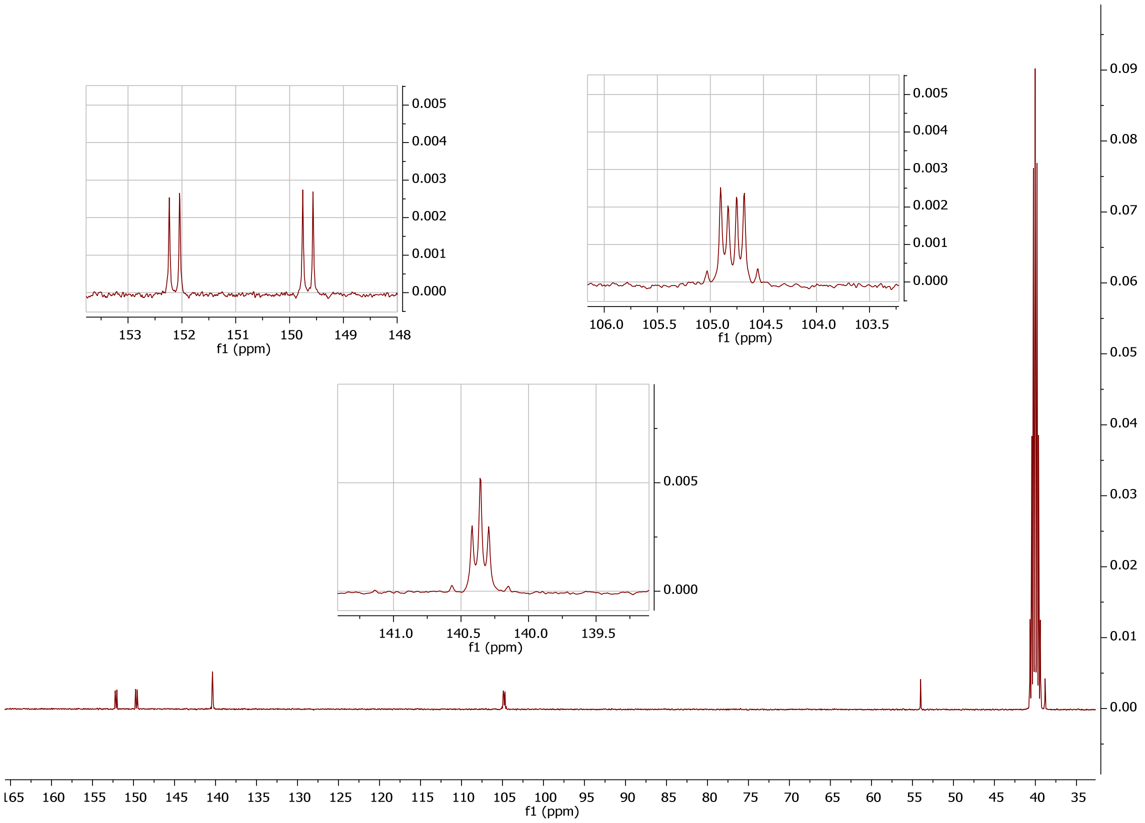

^{13}C NMR spectrum (DMSO at 40 ppm)

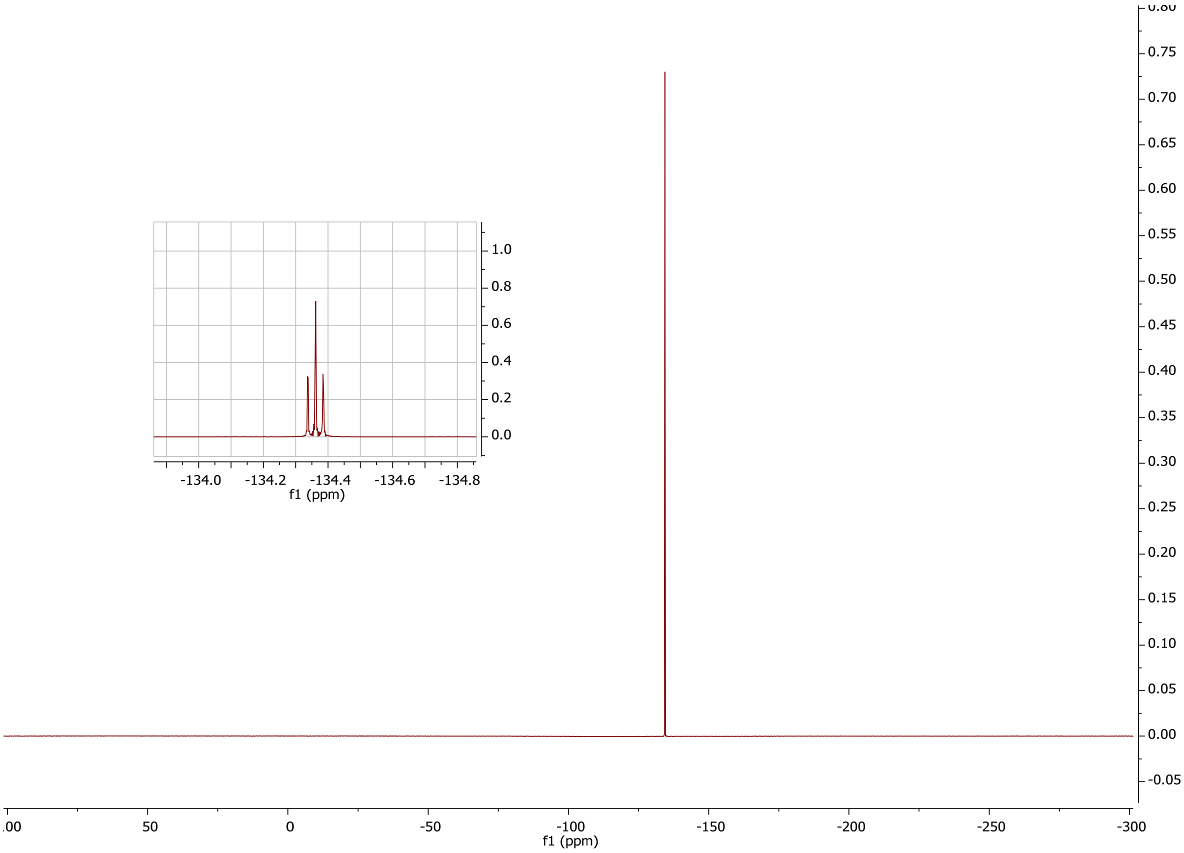

^{19}F NMR spectrum

2. 2D perovskite thin films and single crystals

Materials: The synthesized organic salts were dried under high vacuum and were stored inside a nitrogen-filled glovebox. Lead(II) iodide (99.99%) was purchased from TCI Chemicals. Lead(II) bromide (99.998%) was purchased from Puratronic. Both lead salts were stored inside a nitrogen-filled glovebox. HBr (48% in water) was purchased from Fluorochem. HI (57% in water, distilled, unstabilized), tri-*n*-butyl phosphate (>99%), *N*,*N*-dimethylacetamide (99.5%, dried over molecular sieves) and propylene carbonate (99.5%) were purchased from Acros Organics; all other solvents were purchased from Fisher Scientific.

Thin films fabrication and characterization: Precursor solutions for thin-film synthesis were prepared by dissolving stoichiometric amounts of the corresponding lead halide and organic halide in a mixture of dry *N*,*N*-dimethylformamide (DMF) and dry *N*,*N*-dimethylacetamide (DMAc). The amounts are depicted in Table S1. The precursor elements were dissolved at 50 °C and the resulting solutions were filtered through a syringe filter (0.2 µm pore size). The precursor solutions were spin coated on top of quartz substrates, which had been sonicated in dimethyl sulfoxide, deionized water, acetone, and isopropanol respectively (15 min each) and had been treated by UV/ozone for 15 min. Spin coating and subsequent hotplate annealing were performed inside a nitrogen-filled glovebox (<1 ppm O_2, <1 ppm H_2O). Reference thin-films containing only the organic halide were spin coated by omitting the lead halide in the precursor and by drying at 80 °C after spin coating. The resulting perovskite thin-films were stored inside a nitrogen-filled glovebox and were only removed for analysis.

Table S1: Precursor compositions and spin coating conditions.

<table>
<tr><th></th><th>Organic halide</th><th>Lead halide</th><th>Solvents</th><th>Spin coating</th><th>Annealing (10 min)</th></tr>
<tr><td>$(BTa)_2PbI_4$</td><td>0.30 M BTaI</td><td rowspan="2">0.15 M PbI_2</td><td rowspan="4">DMF/DMAc 9:1 volume ratio</td><td rowspan="3">2000 rpm; 2000 rpm s^{-1}; 20 s</td><td rowspan="2">120 °C</td></tr>
<tr><td>$(F_2BTa)_2PbI_4$</td><td>0.30 M F_2BTaI</td></tr>
<tr><td>$(BTa)_2PbBr_4$</td><td>0.30 M BTaBr</td><td rowspan="2">0.15 M $PbBr_2$</td><td rowspan="2">130 °C</td></tr>
<tr><td>$(F_2BTa)_2PbBr_4$</td><td>0.30 M F_2BTaBr</td><td>4000 rpm; 4000 rpm s^{-1}; 20 s</td></tr>
</table>

UV-vis-NIR absorption spectra were measured on a Cary 5000 UV-Vis-NIR spectrophotometer from Agilent Technologies. A cleaned quartz substrate was used as calibration background. Photoluminescence (PL) emission spectra were measured on a Horiba-Jobin Yvon Fluorolog-3 spectrofluorometer, equipped with double-grating excitation and emission monochromators and a 450 W Xe lamp as a light source. The samples were excited at either 300 nm or 430 nm, which is specified in the relevant figure captions. X-ray diffraction (XRD) measurements were measured at ambient temperature and pressure on a Bruker D8 Discover diffractometer with CuK_α radiation.

Crystallization: Perovskite single-crystals were grown following the solvent conversion-induced crystallization method reported by Fateev et al.[1] Stoichiometric amounts of the organic halide and lead halide were dissolved in a mixture of propylene carbonate (PC), water, and HI (57% in water). HI was extracted three times with a 9:1 v/v mixture of chloroform and tri-*n*-butyl phosphate before use. Lead iodide-based mixtures were stirred at 50 °C and lead bromide-based mixtures were stirred at 40 °C until a clear solution was obtained. The solutions were then filtered through a syringe filter into a base-bath cleaned vial and were heated to their crystallization temperature. The vial lid was equipped with a needle to enable CO_2 to escape

throughout the solvent degradation process. The exact precursor concentration, solvent mixture and crystallization temperature are summarized in Table S2. $(BTa)_2PbI_4$ and $(F_2BTa)_2PbI_4$ crystallized as orange needles (55% and 64% yield respectively); $(BTa)_2PbBr_4$ and $(F_2BTa)_2PbBr_4$ crystallized as colorless platelets (37% and 24% yield respectively).

Table S2: Summary of precursor compositions and crystallization temperatures.

	Organic halide	Lead halide	Solvents	Crystallization temperature
$(BTa)_2PbI_4$	0.29 M BTaI	0.15 M PbI_2	PC/H_2O/HI (57%) 1:1:0.2 molar ratio	50 °C (24 h)
$(F_2BTa)_2PbI_4$	0.60 M F_2BTaI	0.30 M PbI_2		40 ºC (48 h)
$(BTa)_2PbBr_4$	0.17 M BTaBr	0.084 M $PbBr_2$	PC/H_2O/HBr (48%) 1:1:0.2 molar ratio	40 °C (24 h)
$(F_2BTa)_2PbBr_4$	0.11 M F_2BTaBr	0.057 M $PbBr_2$		30 °C (48 h)

X-ray intensity data of single crystals were collected at 100 K on a Rigaku Oxford Diffraction Supernova Dual Source (Cu at zero) diffractometer equipped with an Atlas CCD detector using ω scans and MoK_α (λ = 0.71073 Å) radiation. The images were interpreted and integrated with the program CrysAlisPro.[2] Using Olex2,[3] the structures were solved by direct methods using the ShelXT structure solution program and refined by full-matrix least-squares on F^2 using the ShelXL program package.[4,5] Non-hydrogen atoms were anisotropically refined and the hydrogen atoms in the riding mode and isotropic temperature factors fixed at 1.2 times U(eq) of the parent atoms.

Crystal data for $(BTa)_2PbI_4$: $C_{16}H_{22}I_4N_8Pb$; MW = 1041.22; orthorhombic, space group *Pnma* (No. 62); a = 12.8383(2) Å, b = 29.6464(4) Å, c = 6.5400(1) Å, α = 90°, β = 90°, γ = 90°; V =

2489.19(6) Å^3; Z = 4; ρ = 2.778 g cm^{-3}; F(000) = 1872; R_gt = 0.0254 (3156 reflections), wR_ref = 0.0614 (3422 reflections).

Crystal data for $(F_2BTa)_2PbI_4$: $C_{16}H_{18}F_4I_4N_8Pb$; MW = 1113.18; orthorhombic, space group *Pnma* (No. 62); a = 12.8517(2) Å, b = 29.9017(5) Å, c = 6.57340(10) Å, α = 90°, β = 90°, γ = 90°; V = 2526.08(7) Å^3; Z = 4; ρ = 2.927 g cm^{-3}; F(000) = 2000; R_gt = 0.0210 (3025 reflections), wR_ref = 0.0432 (3350 reflections).

Crystal data for $(BTa)_2PbBr_4$: $C_{16}H_{22}Br_4N_8Pb$; MW = 853.22; monoclinic, space group *C2/c* (No. 15); a = 37.1774(5) Å, b = 5.71880(10) Å, c = 11.36340(10) Å, α = 90°, β = 97.1010(10)°, γ = 90°; V = 2397.44(6) Å^3; Z = 4; ρ = 2.364 g cm^{-3}; F(000) = 1584; R_gt = 0.0187 (3127 reflections), wR_ref = 0.0460 (3265 reflections).

Crystal data for $(F_2BTa)_2PbBr_4$: $C_{16}H_{18}Br_4F_4N_8Pb$; MW = 925.18; monoclinic, space group *$P2_1/c$* (No. 14); a = 18.6625(5) Å, b = 11.5643(3) Å, c = 11.5797(3) Å, α = 90°, β = 99.385(2)°, γ = 90°; V = 2465.67(11) Å^3; Z = 4; ρ = 2.492 g cm^{-3}; F(000) = 1712; R_gt = 0.0361 (4814 reflections), wR_ref = 0.0726 (6236 reflections).

CCDC 2228611-2228614 contain the supplementary crystallographic data for this paper. These data can be obtained free of charge from The Cambridge Crystallographic Data Centre via www.ccdc.cam.ac.uk/structures.

3. $FAPbI_3$ perovskite solar cells

Materials: The colloidal SnO_2 dispersion (Alfa Aesar), [6,6]-phenyl-C_{61}-butyric acid (PCBA), PbI_2 (TCI Chemicals, 99.99%), formamidinium iodide (FAI) (GreatCell Solar, >99.99%), methylammonium chloride (MACl) (GreatCell Solar, >99.99%), 2,2',7,7'-tetrakis[*N*,*N*-di(4-methoxyphenyl)amino]-9,9'-spirobifluorene (Spiro-OMeTAD) (Lumtec), tert-butylpyridine (Sigma, 98%), lithium bis(trifluoromethanesulfonyl)imide (Li-TFSI) (Sigma-Aldrich, 99.95% trace metal basis) and tris(2-(1H-pyrazol-1-yl)-4-tert-butylpyridine) cobalt(III)

tri[bis(trifluoromethane) sulfonimide] (Co-TFSI) (Sigma, 98%), DMF (Sigma, anhydrous 99.8%), dimethyl sulfoxide (DMSO) (Sigma, anhydrous 99.9%), *o*-dichlorobenzene (Sigma, anhydrous 99%), and 2-propanol (Sigma, anhydrous 99.8%), were obtained from commercial sources as indicated and used without further purification.

Solutions preparation: The SnO_2 colloidal dispersion was diluted with 5 parts H_2O. PCBA was dissolved in *o*-dichlorobenzene (0.2 mg mL^{-1}). PbI_2 (691.5 mg) was dissolved in DMF/DMSO 9:1 (v/v) (1 mL). FAI (90 mg) and MACl (9 mg) were dissolved in 2-propanol (1 mL). BTaX solutions were prepared by dissolving the salts in 2-propanol at concentrations of 1 or 10 mg mL^{-1}. Li-TFSI and Co-TFSI were dissolved in acetonitrile at concentrations of 500 mg mL^{-1}. Spiro-OMeTAD (40 mg) was dissolved in *o*-chlorobenzene (0.5 mL) to which tert-butylpyridine (14.3 μL) and Li-TFSI in acetonitrile (8.8 μL) were added, followed by addition of Co-TFSI in acetonitrile (10 μL) just before spin coating.

Device fabrication: ITO was sputtered onto glass substrates with a thickness of 90 nm. Substrates were then cleaned by sonication in acetone (15 min), and sodium dodecyl sulfate (99%, Acros) soapy water (15 min), rinsed with deionized water, and then sonicated again in 2-propanol (15 min). Before spin coating, substrates were treated with UV-ozone for 30 min. SnO_2 was spin coated at 2800 rpm for 60 s with 2000 rpm s^{-1} acceleration and annealed at 150 °C for 30 min. ITO/SnO_2 substrates were then transferred to an N_2-filled glovebox after treating them with UV-ozone for 15 min. PCBA was spin coated at 2000 rpm for 30 s with 2000 rpm s^{-1} acceleration and no annealing. PbI_2 was spin coated at 1500 rpm for 30 s with 3000 rpm s^{-1} acceleration and annealed at 70 °C for 1 min. After cooling down, FAI/MACl were spin coated onto PbI_2 layer at 2000 rpm for 30 s with 20,000 rpm s^{-1} acceleration and annealed at 150 °C for 1 min. The passivation layer was dynamically spin coated at 5000 rpm for 45 s. Afterwards, substrates were brought in ambient air and annealed at 150 °C for 15 min. Spiro-OMeTAD was spin coated at 2000 rpm for 50 s with 20,000 rpm s^{-1} acceleration. No annealing was performed.

The layers were then doped in air for 20 min. 10 nm MoO_3 and 100 nm Au were thermally evaporated in high vacuum. 100 nm MgF_2 was thermally evaporated on the glass side as anti-reflective coating.

J−V characterization: A tungsten-halogen lamp combined with a daylight filter (Hoya LB120) and a UV-filter (Schott GG385) were used to simulate the solar spectrum. The light intensity was calibrated via a Si photodiode. The solar cell area was defined by a black shadow mask with an aperture of 0.0676 cm^2 or 0.1296 cm^2. *J−V* measurements were performed with a Keithley 2400 source meter to sweep the voltage from −0.5 V (1.5 V) to 1.5 V (−0.5 V) at a scan rate of 0.25 V s^{-1} in both reverse and forward scan. Stability measurements were performed in an N_2-filled glovebox by keeping the devices at the maximum power point voltage under 1-sun illumination and tracking the photocurrent over time.

EQE characterization: For EQE measurements, a modulated (Oriel, Cornerstone 130) tungsten-halogen lamp (Philips Focusline, 50 W) was used light source. The signal was amplified via a current preamplifier (Stanford Research, SR 570) and measured by a lock-in amplifier (Stanford Research, SR 830). Based on a calibrated silicon reference cell, the EQE is then calculated from the spectral response signal. An additional 530 nm LED (Thorlabs) was used to generate a photocurrent in the solar cell during the EQE measurement, which is close to the current measured during *J−V* measurements, to mimic 1-sun condition.

***Quasi-Fermi Level Splitting*:** Absolute photoluminescence spectroscopy was performed using a 455 nm LED (Thorlabs, M455F3-455 fiber coupled LED) source to excite the perovskite film through an optical fiber placed in an integrating sphere (Avantes, AvaSphere-30-REFL) fitted with a 550 nm short-pass filter (Edmund Optics). The excitation intensity was calibrated to 1-sun equivalent intensity. The spectrum was measured through an optical fiber connected to a calibrated AvaSpec-HERO spectrometer (Avantes, AVASPEC- HSC1024X58TEC-EVO) using a 550 nm long-pass filter.

Optical characterization: PL spectra were recorded by using an Edinburgh Instruments FLSP920 double-monochromator luminescence spectrophotometer.

X-Ray characterization: XRD patterns were recorded by using a Bruker 2D phaser (CuK_{α} radiation, $\lambda = 1.5406$ Å): measurements were performed in the range 3-40° with a step size 0.02° and collection time of 1 s. 2D GIWAXS measurements were performed with a Ganesha 300XL+ system from JJ X-ray equipped with a Pilatus 300K detector (pixel side 172 μm × 172 μm). The X-ray source was a Genix 3D Microfocus sealed tube X-ray Cu-source with integrated monochromator. The wavelength used was 1.5408 Å. The detector is in a vacuum chamber with sample-to-detector distance (SDD) of 515.4 mm, as calibrated using silver behenate (d_{001} = 58.380 Å). The angle-dependent 2D GIWAXS was conducted via controlling incidence angle from 0.1° to 0.5° with 0.1° interval. XPS measurements were performed using a Thermo Scientific K-Alpha with a 180° double focusing hemispherical analyzer and a 128-channel detector. Monochromatic Al Kα (1486.6 eV) radiation was used, and the X-ray spot size was 400 μm. For the surface analysis, a survey spectrum was first measured for 12 scans with a pass energy of 200 eV. High-resolution scan (20 times) of each element was conducted with a pass energy of 50 eV. During the sputtering experiment, the sample was removed layer-by-layer by argon ion etching operated at low current and low ion energy (1000 eV). The sputtering speed corresponds to 0.012 nm s^{-1}. The crater region generated by argon ions is ~2 × 4 mm^2. For the depth profiles, snapshot mode was used for each element, and the number of frames was 5 × 1 s. For the depth profile of N 1*s*, high-resolution scans were used instead of snapshots (20 scans).

SEM and CL measurements: SEM images for Figure S10-11 were measured with a FEI Quanta 3D FEG microscope (5 keV electron beam, secondary electron detector). CL hyperspectral maps from SEM were acquired in an Attolight Allalin 4027 Chronos in continuous wave mode. Spectra were acquired with an iHR320 spectrometer (focal length of 320 mm, 150 gratings per mm blazed at 500 nm, 7000 μm entrance slit) coupled to an Andor 1024 px charge-coupled

device (readout rate of 50 kHz, and 4× signal amplification). Measurements were performed at room temperature under a high vacuum. To prevent specimen damage before the measurement, beam focusing was performed away from the areas of interest. All maps were acquired at 3 kV acceleration voltage, 62.5 pA beam current, at 128x128 px resolution in 5.5-μm field of view and 23.8 ms/px acquisition time. These conditions were found to be the optimum balance between electron beam damage and spatial resolution. SE images were acquired after the hyperspectral mapping, at 1024×1024 px resolution and 10 μs/px dwell time. CL maps were background subtracted, processed, and analyzed in LumiSpy 0.2.[6] The artefacts caused by cosmic rays saturating the spectrometer were removed, and the edges of each map were cropped out as they tend to show edge effects and higher CL intensities. Energy bandpass-filtered images were formed by integrating CL intensity over specific energy ranges.

Additional Figures

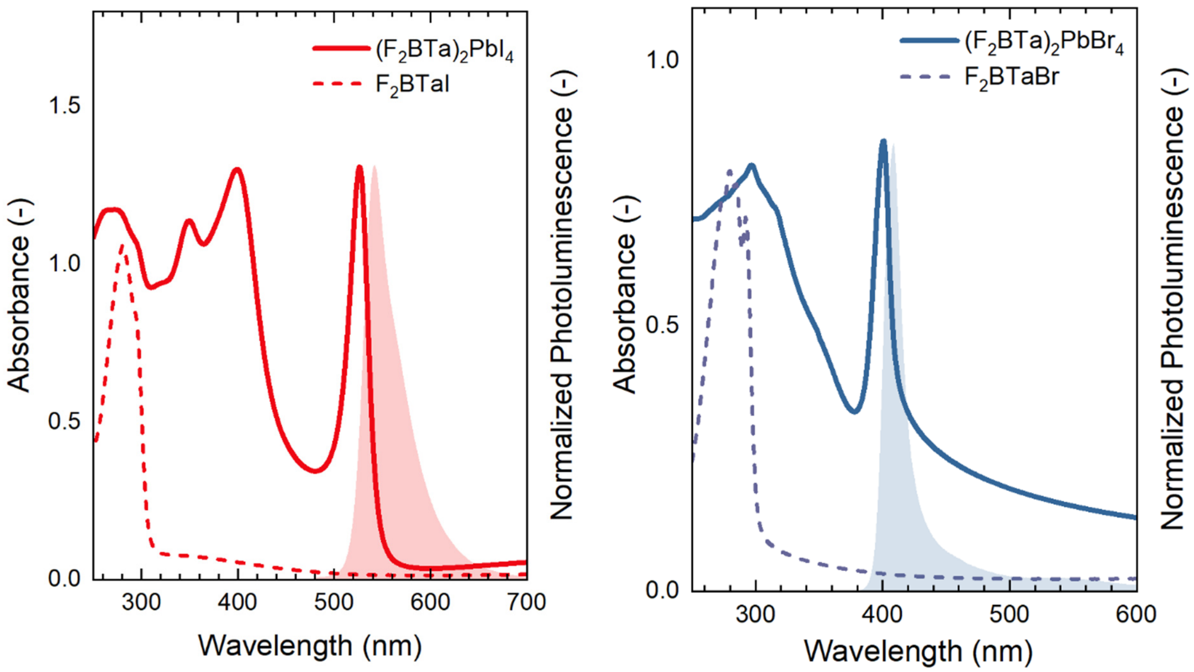

Figure S1. UV-vis and PL spectra of $(F_2BTa)_2PbI_4$ (left) and $(F_2BTa)_2PbBr_4$ (right). Solid and dashed lines represent UV-vis absorption of the perovskite film and pure benzotriazole salt, respectively, whereas shaded area represents PL of the perovskite film. PL spectra were obtained with excitation at 430 and 300 nm, respectively.

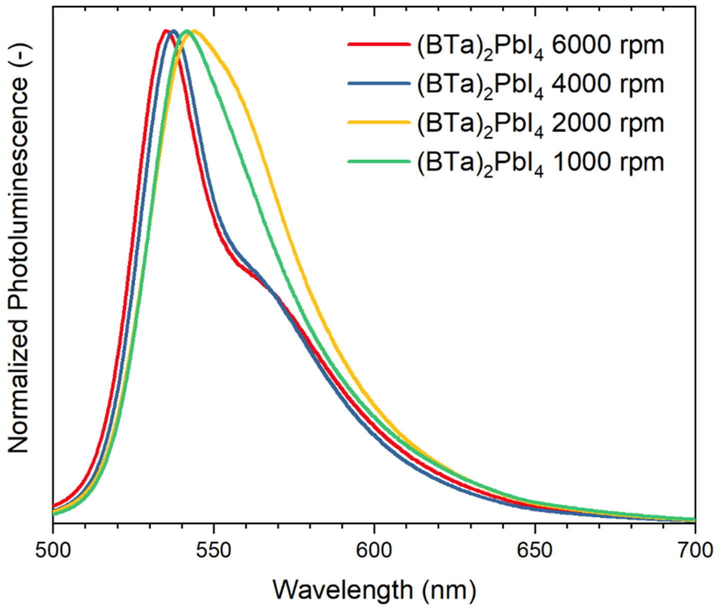

Figure S2. PL spectra of $(BTa)_2PbI_4$ films processed at different spin coating speeds to achieve different film thickness.

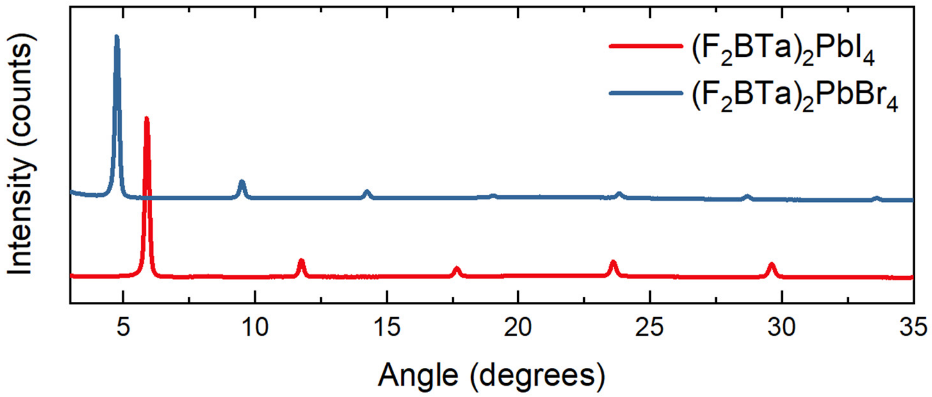

Figure S3. XRD diffraction patterns of $(F_2BTa)_2PbI_4$ and $(F_2BTa)_2PbBr_4$ thin films.

(a)

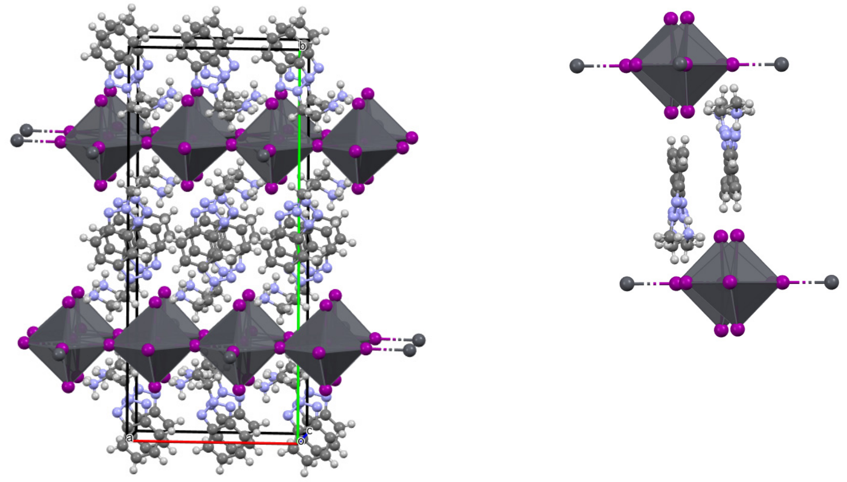

(b)

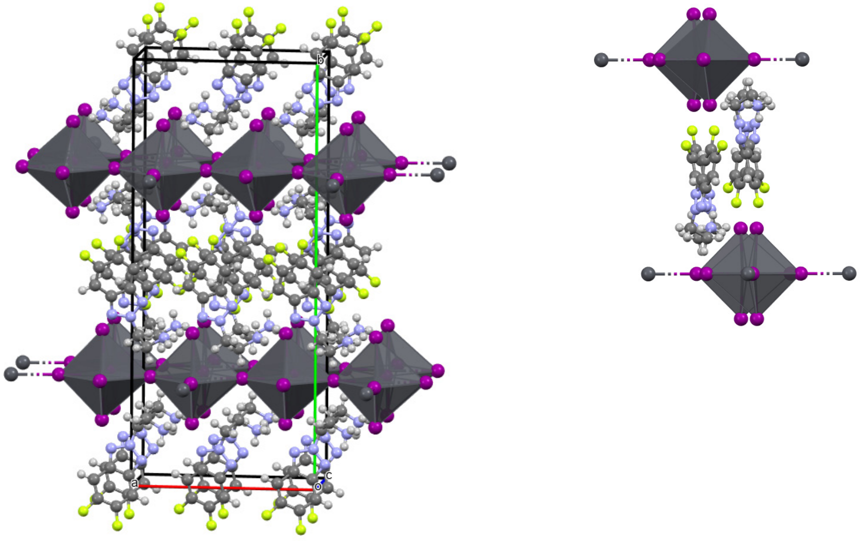

(c)

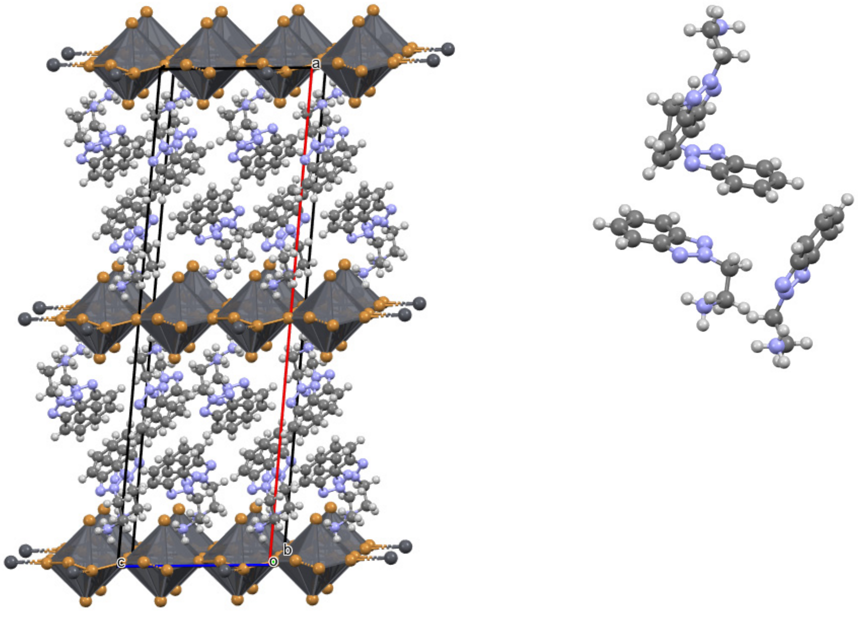

(d)

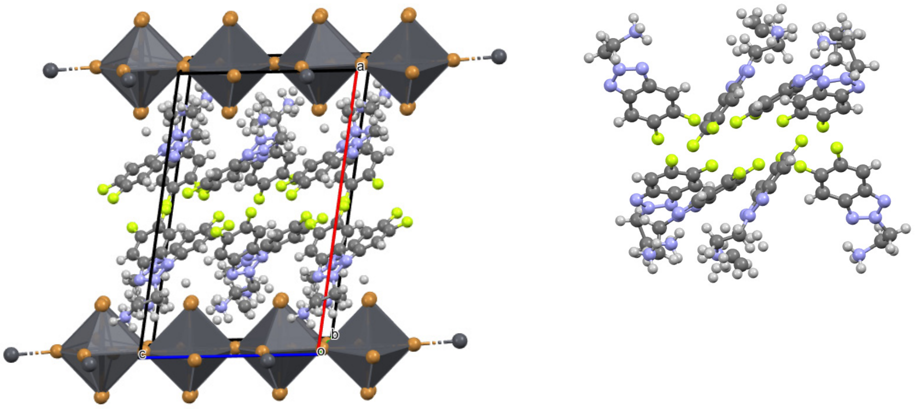

Figure S4. Packing arrangements of benzotriazole-based single crystals. The images on the left contain the unit cell of each arrangement; the images on the right show a detail of the packing of the organic layers. (a) $(BTa)_2PbI_4$. (b) $(F_2BTa)_2PbI_4$. (c) $(BTa)_2PbBr_4$. (d) $(F_2BTa)_2PbBr_4$. Color codes: black (Pb), purple (I), brown (Br), grey (C), white (H), blue (N), and yellow (F). Spacings (d) are: (a) $b/2$ = 14.8 Å, (b) $b/2$ = 15.0 Å, (c) $a/2$ = 18.6 Å, (d) a = 18.7 Å.

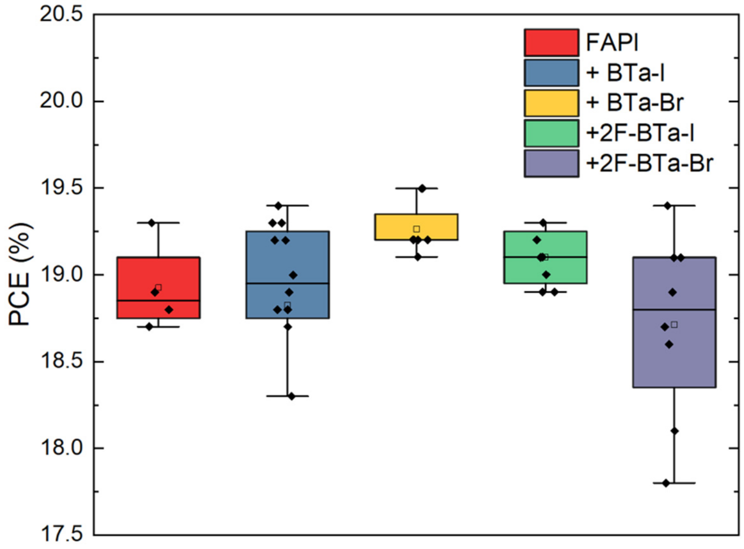

Figure S5. PCE boxplot of $ITO/SnO_2/PCBA/FAPbI_3/Spiro\text{-}OMeTAD/MoO_3/Au$ solar cells in which the $FAPbI_3$ layer is passivated with different BTa derivatives. The box represents the interquartile range and the whiskers represent the minimum and maximum measured value.

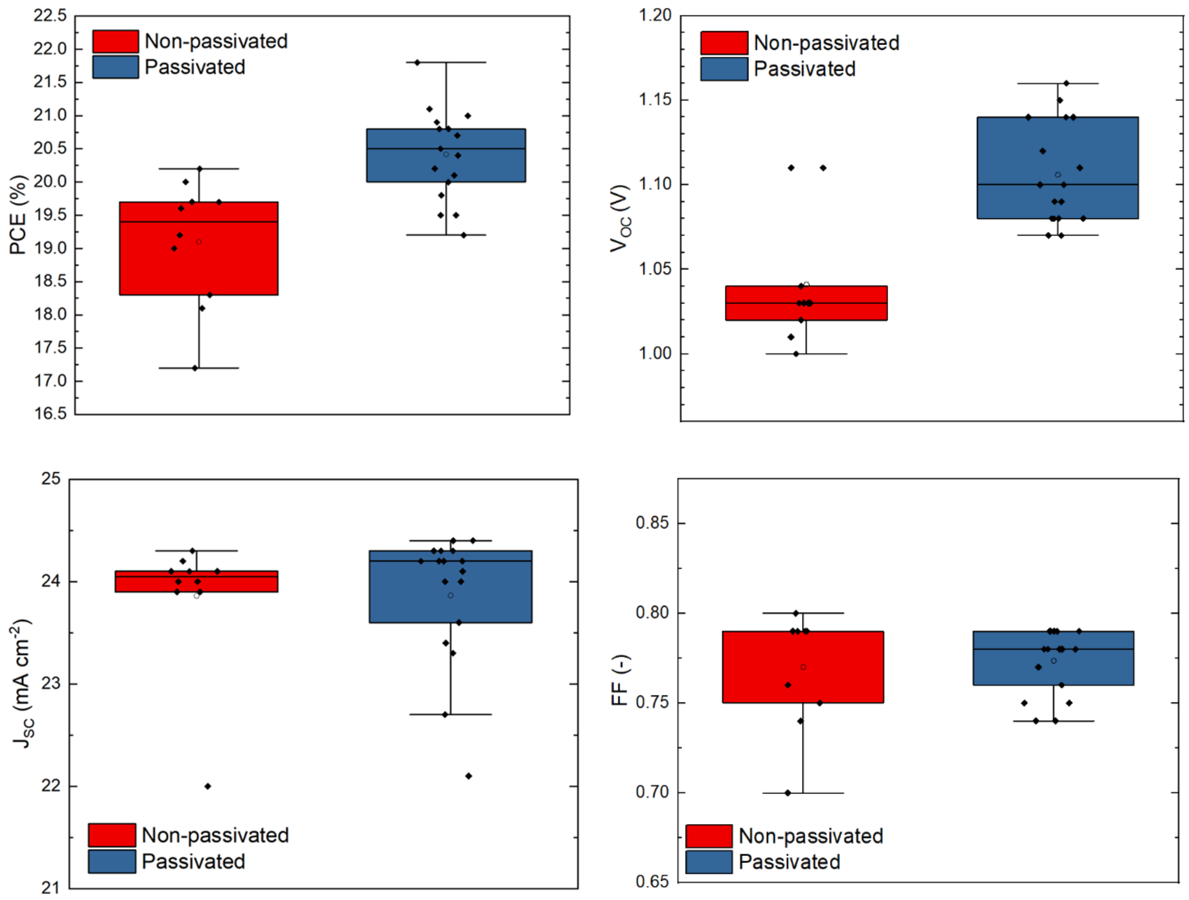

Figure S6. Boxplots for PCE V_{OC}, J_{SC}, and FF of ITO/SnO_2/PCBA/$FAPbI_3$/Spiro-OMeTAD/MoO_3/Au solar cells without and with passivation of $FAPbI_3$ with BTaBr (1 mg mL^{-1}). The box represents the interquartile range and the whiskers represent the minimum and maximum measured value.

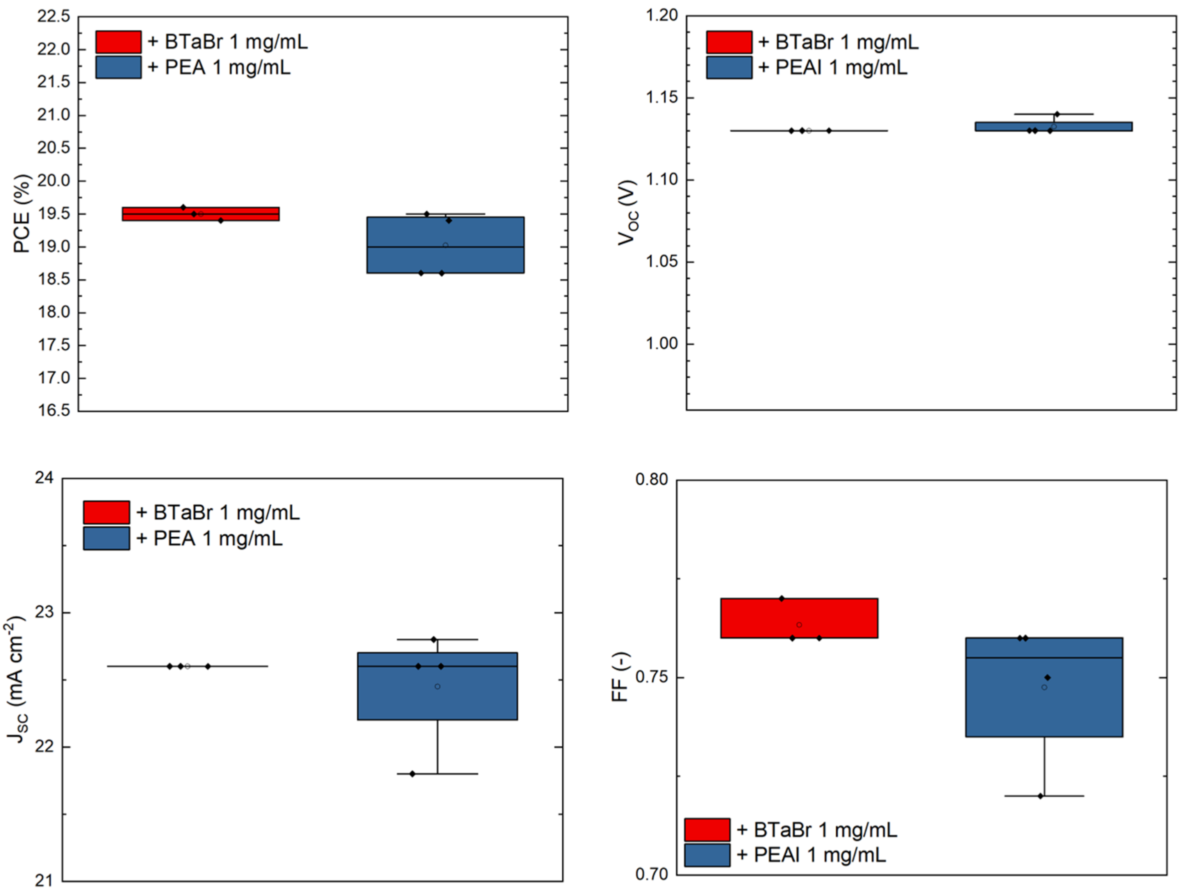

Figure S7. Boxplots for PCE V_{OC}, J_{SC}, and FF of ITO/SnO_2/PCBA/$FAPbI_3$/Spiro-OMeTAD/MoO_3/Au solar cells, passivated by BTaBr (1 mg mL^{-1}) or PEAI (1 mg mL^{-1}). The box represents the interquartile range and the whiskers represent the minimum and maximum measured value.

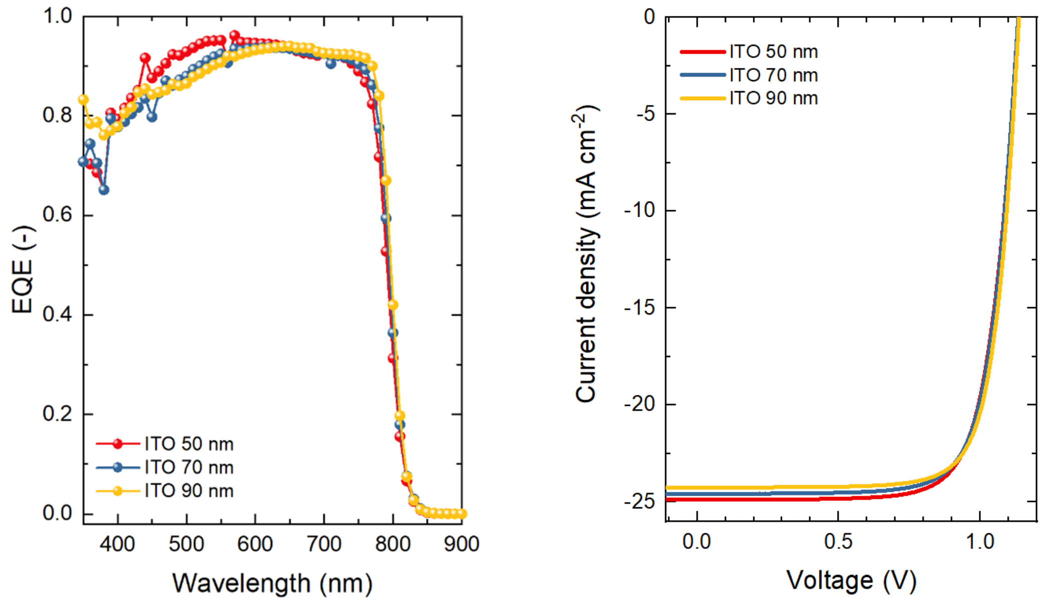

Figure S8. ITO/SnO_2/PCBA/$FAPbI_3$/Spiro-OMeTAD/MoO_3/Au solar cells with varying thickness of the ITO bottom electrode. (left) EQE spectra. (right) *J*−*V* curves. By changing the ITO thickness, going from 90 nm (reference device) to 50 nm, we could enhance the EQE in the abovementioned region, although the higher sheet resistance of ITO led to a slight FF loss and overall comparable performances with the reference. Higher J_{SC} values are also achievable by increasing the perovskite layer thickness, although we found that morphology issues, such as increased roughness of the active layer, become significant and lead to performance losses.

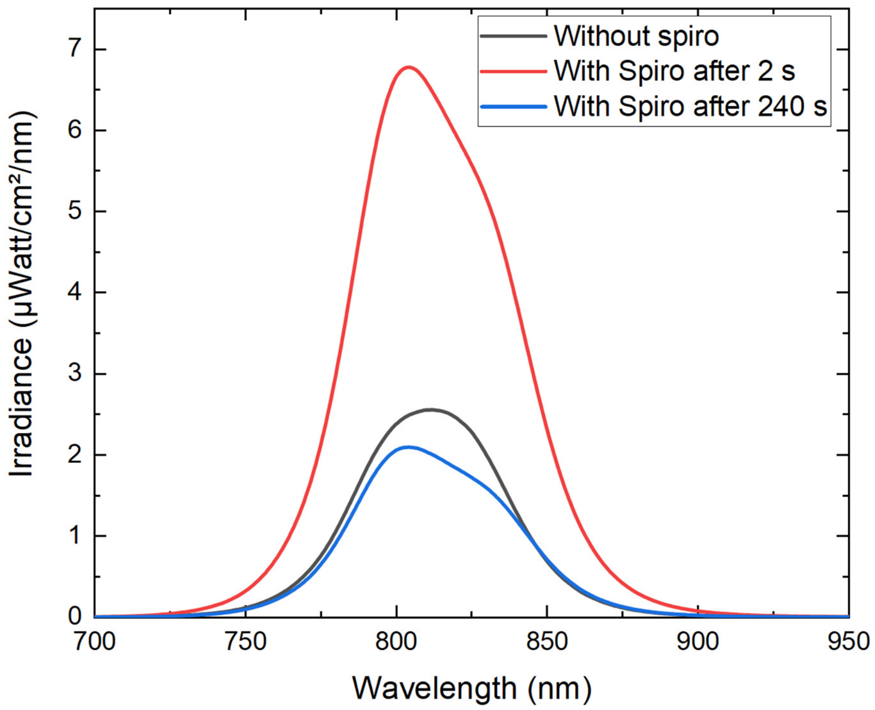

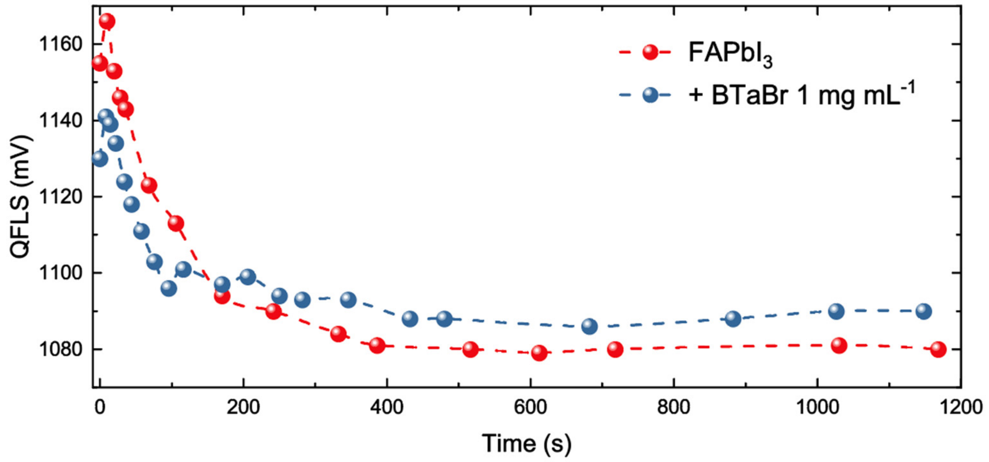

Figure S9. Absolute PL spectra for $FAPbI_3$/Spiro-OMeTAD layers recorded at different times after starting the measurement (top). QFLS tracking of $FAPbI_3$/Spiro and $FAPbI_3$/2D/Spiro films (bottom).

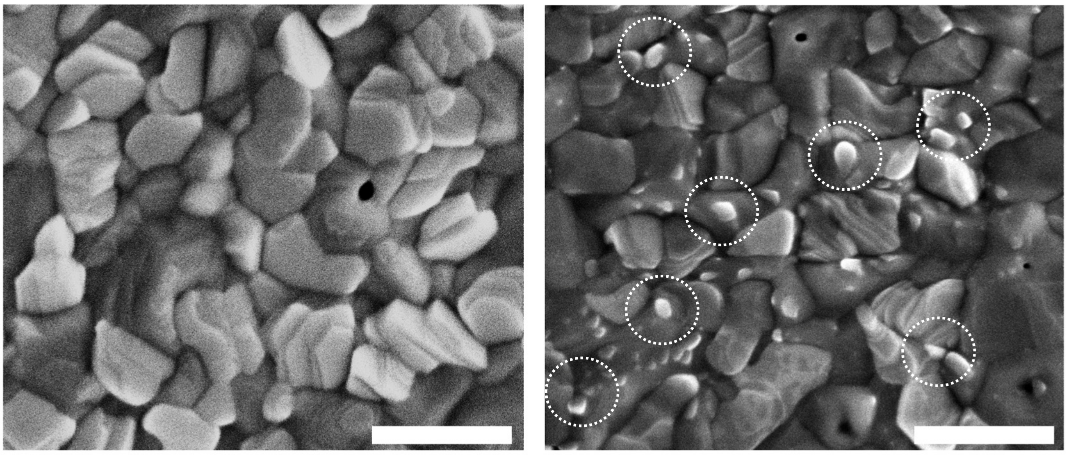

Figure S10. SEM images of $FAPbI_3$ before (left) and after (right) passivation with BTaBr 1 mg mL^{-1}. New crystallites appear on the surface after passivation. Scalebar is 1 μm.

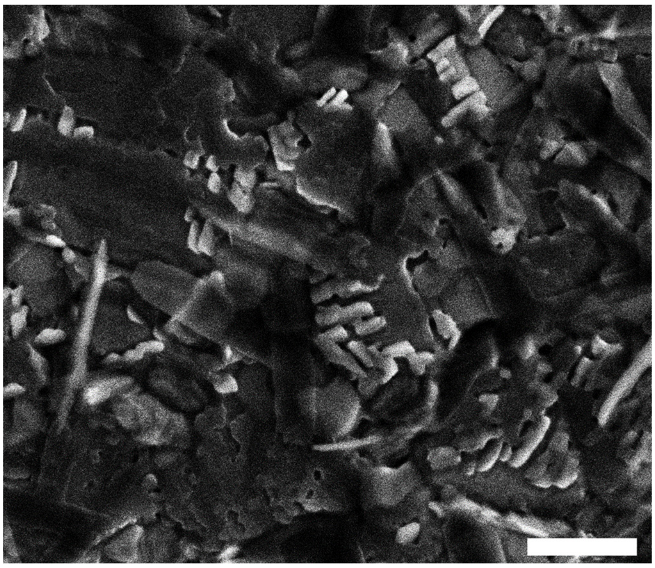

Figure S11. SEM images of $FAPbI_3$ after passivation with BTaBr 10 mg mL^{-1}. Scalebar is 1 μm.

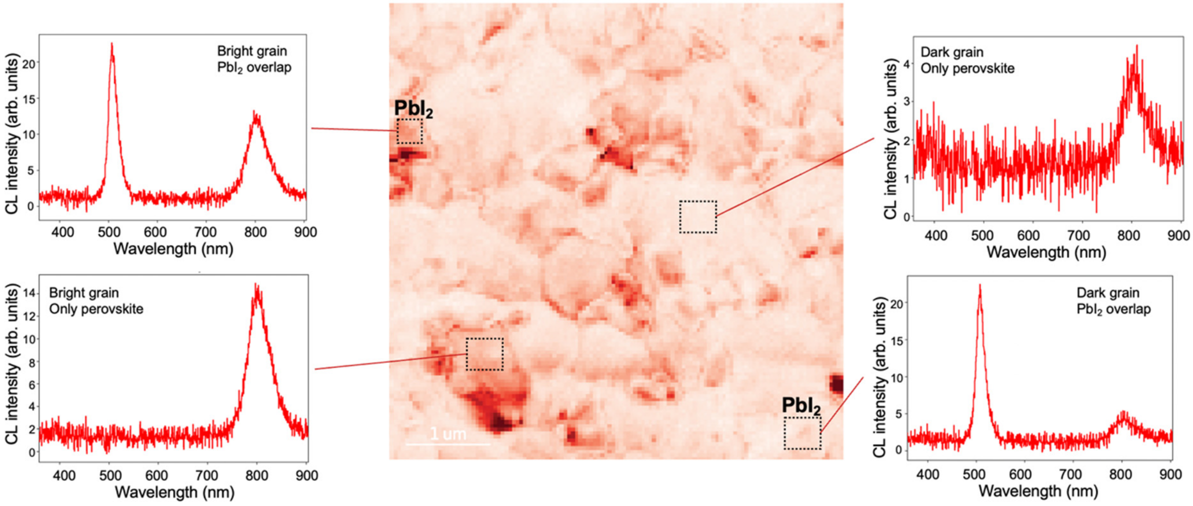

Figure S12. SEM-CL image of $FAPbI_3$ (CL emission 800 nm ± 10 nm) with correspondent CL spectra. Highlighted regions were selected to analyze emissive (bright) and non-emissive (dark) grains in presence or absence of PbI_2.

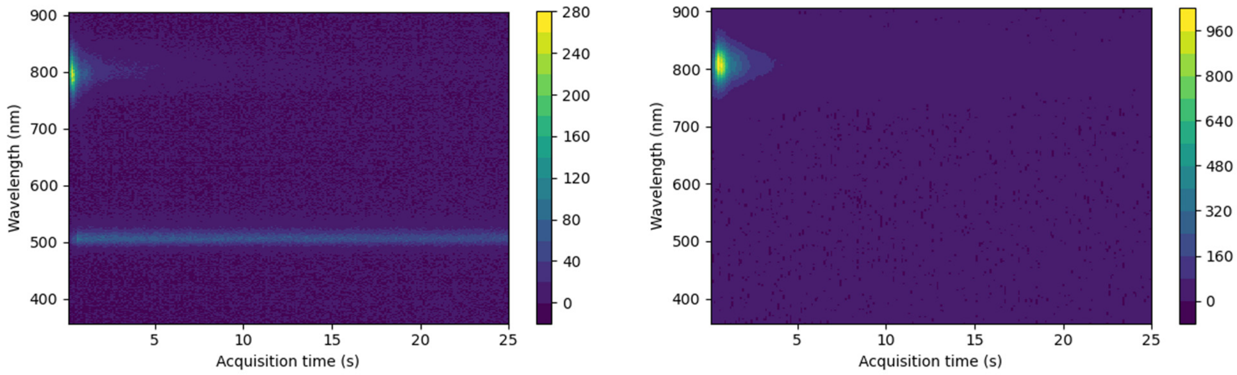

Figure S13. Sample stability over electron beam exposure for $FAPbI_3$ (left) and $FAPbI_3$ + BTaBr 1 mg mL^{-1} (right). Passivated sample shows ~3x CL intensity compared to the non-passivated sample.

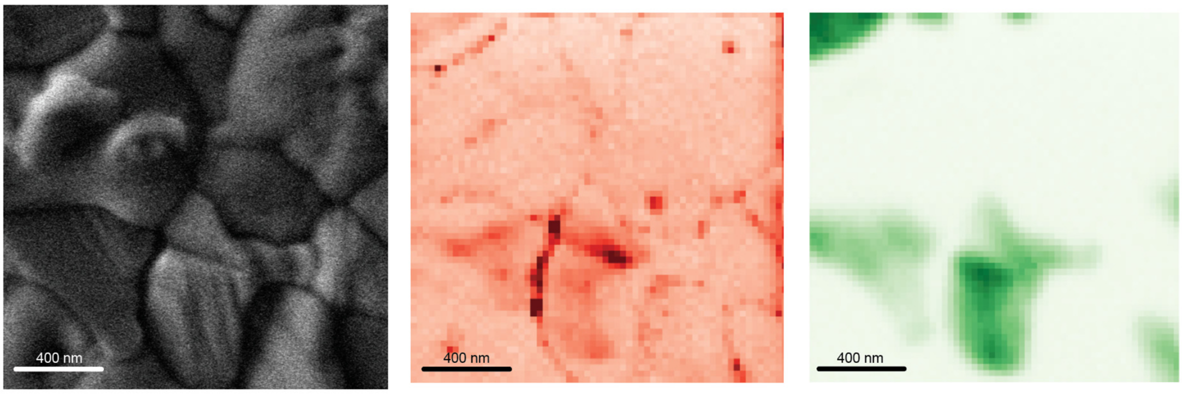

Figure S14. SEM-CL image of $FAPbI_3$ + BTaBr 1 mg mL^{-1} at high magnification: SEM image (left), perovskite emission ~800 nm (center), PbI_2 emission ~520 nm (right).

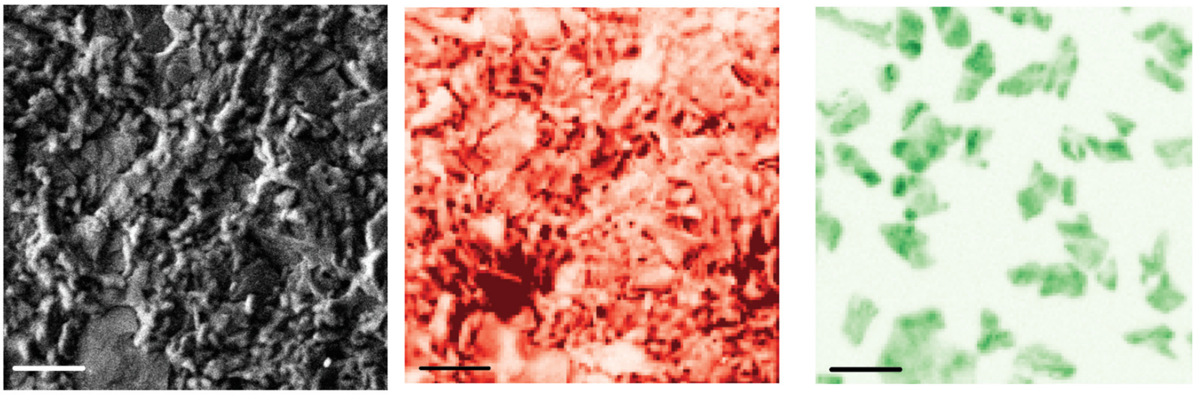

Figure S15. SEM-CL image of $FAPbI_3$ + BTaBr 10 mg mL^{-1}: SEM image (left), perovskite emission ~800 nm (center), PbI_2 emission ~520 nm (right). Scalebars are 1 μm.

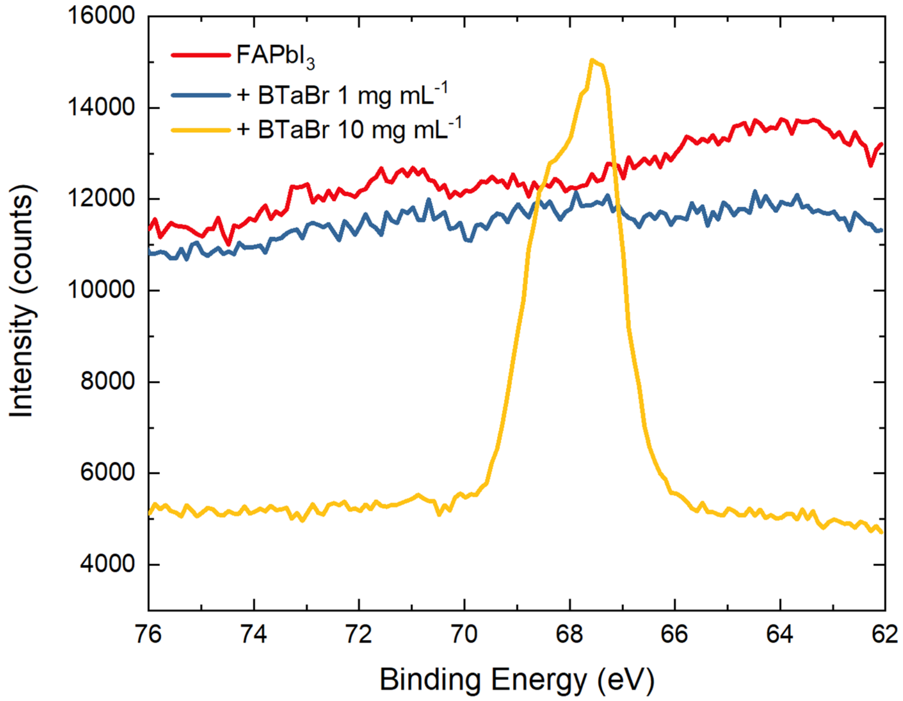

Figure S16. Br 3*s* XPS spectra for $FAPbI_3$, + BTaBr 1 mg mL^{-1}, and + BTaBr 10 mg mL^{-1}. Atomic concentration of Br is 1.2, 0.7, 5.7 at%, respectively. The first two values fall into noise levels.

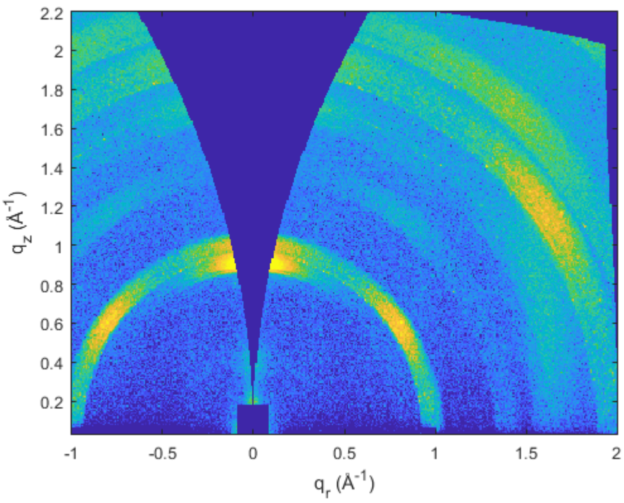

Figure S17. GIWAXS pattern for $FAPbI_3$ + BTaBr 1 mg mL^{-1}.

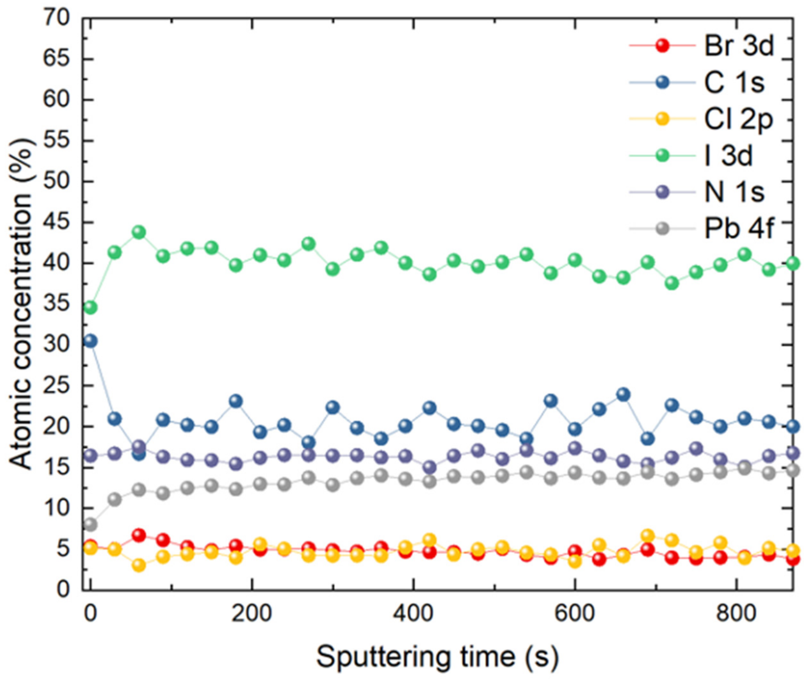

Figure S18. Atomic concentrations of $FAPbI_3$ determined from the XPS depth profiling with Ar ion sputtering. Sputtering cycles = 30 (30 s each).

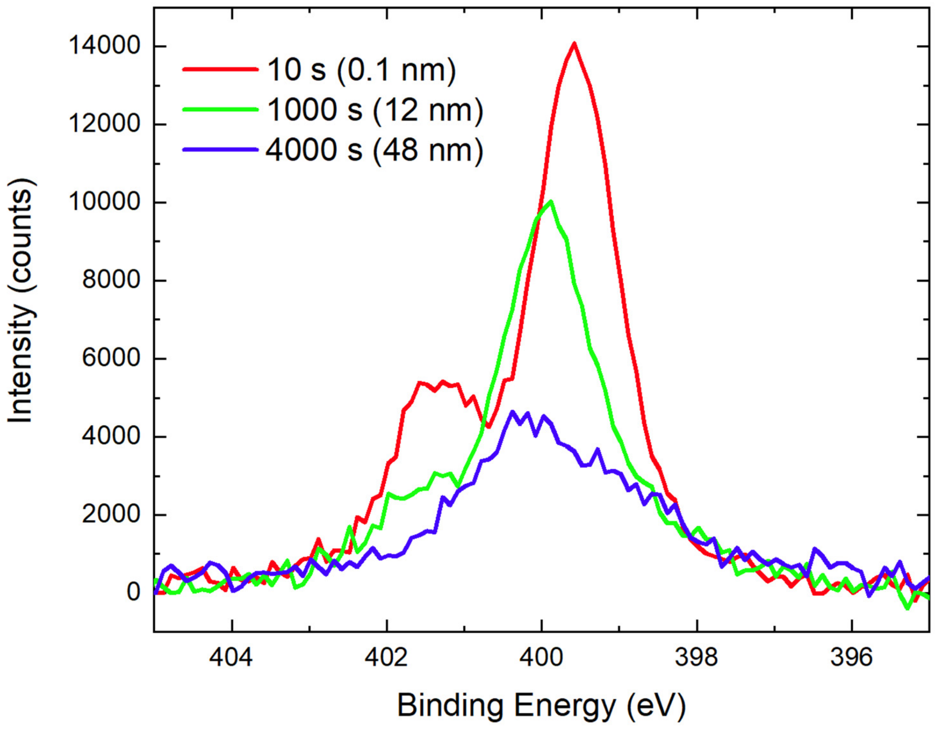

Figure S19. Representative N 1*s* XPS spectra at 10, 1000, and 4000 s Ar ion sputtering time. The three spectra are representative of the three regimes of interlayer removal.

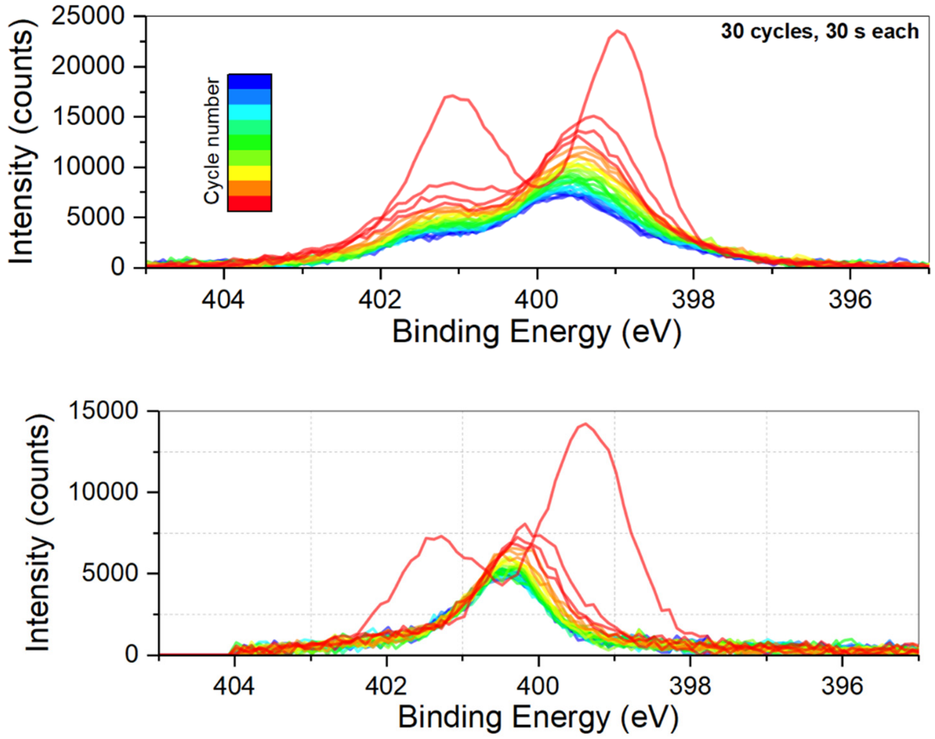

Figure S20. Depth-profile N 1*s* XPS spectra for $FAPbI_3$ + BTaBr 10 (top) and 1 (bottom) mg mL^{-1}. 30 cycles (30 s sputtering each). With these sputtering conditions, the two N 1*s* peaks are still visible for the film passivated with concentrated BTaBr, whereas they disappear after only one cycle for the diluted BTaBr.

Additional references

1. Fateev, S. A.; Petrov, A. A.; Ordinartsev, A. A.; Grishko, A. Y.; Goodilin, E. A.; Tarasov, A. B. *Chem Mater* **2020**, *32*, 9805.
2. CrysAlis Pro, *Rigaku Oxford Diffraction*, Yarnton, England, **2015**.
3. Dolomanov, O. V.; Bourhis, R L.; Gildea, J. J.; Howard, J. A. K.; Puschmann, H. *J. Appl. Crystallogr.* **2009**, *42*, 339.
4. Sheldrick, G. M. *Acta Crystallogr. Sect. A Found. Adv.* **2015**, *71*, 3.
5. Sheldrick, G. M. *Acta Crystallogr. Sect. C Struct. Chem.* **2015**, *71*, 3.
6. https://zenodo.org/record/5233163#.YWmGCRrMKUk
